# Highly Controllable and Silicon-Compatible Ferroelectric Photovoltaic Synapses for Neuromorphic Computing

**DOI:** 10.1016/j.isci.2020.101874

**Published:** 2020-11-30

**Authors:** Shengliang Cheng, Zhen Fan, Jingjing Rao, Lanqing Hong, Qicheng Huang, Ruiqiang Tao, Zhipeng Hou, Minghui Qin, Min Zeng, Xubing Lu, Guofu Zhou, Guoliang Yuan, Xingsen Gao, Jun-Ming Liu

**Affiliations:** 1 Institute for Advanced Materials, South China Academy of Advanced Optoelectronics, South China Normal University, Guangzhou 510006, China; 2 Guangdong Provincial Key Laboratory of Optical Information Materials and Technology, South China Academy of Advanced Optoelectronics, South China Normal University, Guangzhou 510006, China; 3 Department of Industrial Systems Engineering and Management, National University of Singapore, 117576, Singapore; 4 National Center for International Research on Green Optoelectronics, South China Normal University, Guangzhou 510006, China; 5 School of Materials Science and Engineering, Nanjing University of Science and Technology, Nanjing 210094, China; 6 Laboratory of Solid State Microstructures and Innovation Center of Advanced Microstructures, Nanjing University, Nanjing 210093, China

**Keywords:** Circuit Systems, Electrical Engineering, Semiconductor Manufacturing, Materials Science, Devices

## Abstract

Ferroelectric synapses using polarization switching (a purely electronic switching process) to induce analog conductance change have attracted considerable interest. Here, we propose ferroelectric photovoltaic (FePV) synapses that use polarization-controlled photocurrent as the readout and thus have no limitations on the forms and thicknesses of the constituent ferroelectric and electrode materials. This not only makes FePV synapses easy to fabricate but also reduces the depolarization effect and hence enhances the polarization controllability. As a proof-of-concept implementation, a Pt/Pb(Zr_0.2_Ti_0.8_)O_3_/LaNiO_3_ FePV synapse is facilely grown on a silicon substrate, which demonstrates continuous photovoltaic response modulation with good controllability (small nonlinearity and write noise) enabled by gradual polarization switching. Using photovoltaic response as synaptic weight, this device exhibits versatile synaptic functions including long-term potentiation/depression and spike-timing-dependent plasticity. A simulated FePV synapse-based neural network achieves high accuracies (>93%) for image recognition. This study paves a new way toward highly controllable and silicon-compatible synapses for neuromorphic computing.

## Highlights

• Switchable ferroelectric photovoltaic (FePV) effect is used for synaptic application
• Tunable photovoltaic response is enabled by gradual polarization switching
• Versatile synaptic functions and high image recognition accuracy are achieved
• The FePV synapses are facilely grown on silicon substrates

## Graphical Abstract

**Figure:**
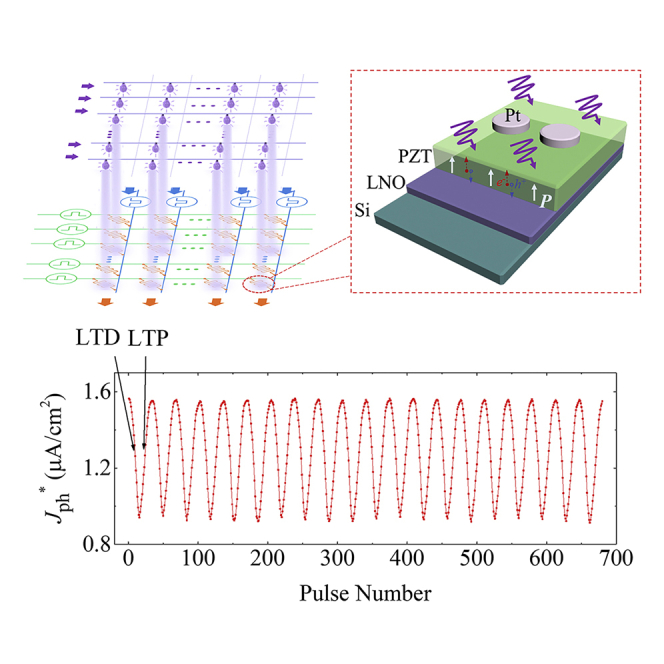

## Introduction

**Figure 1:**
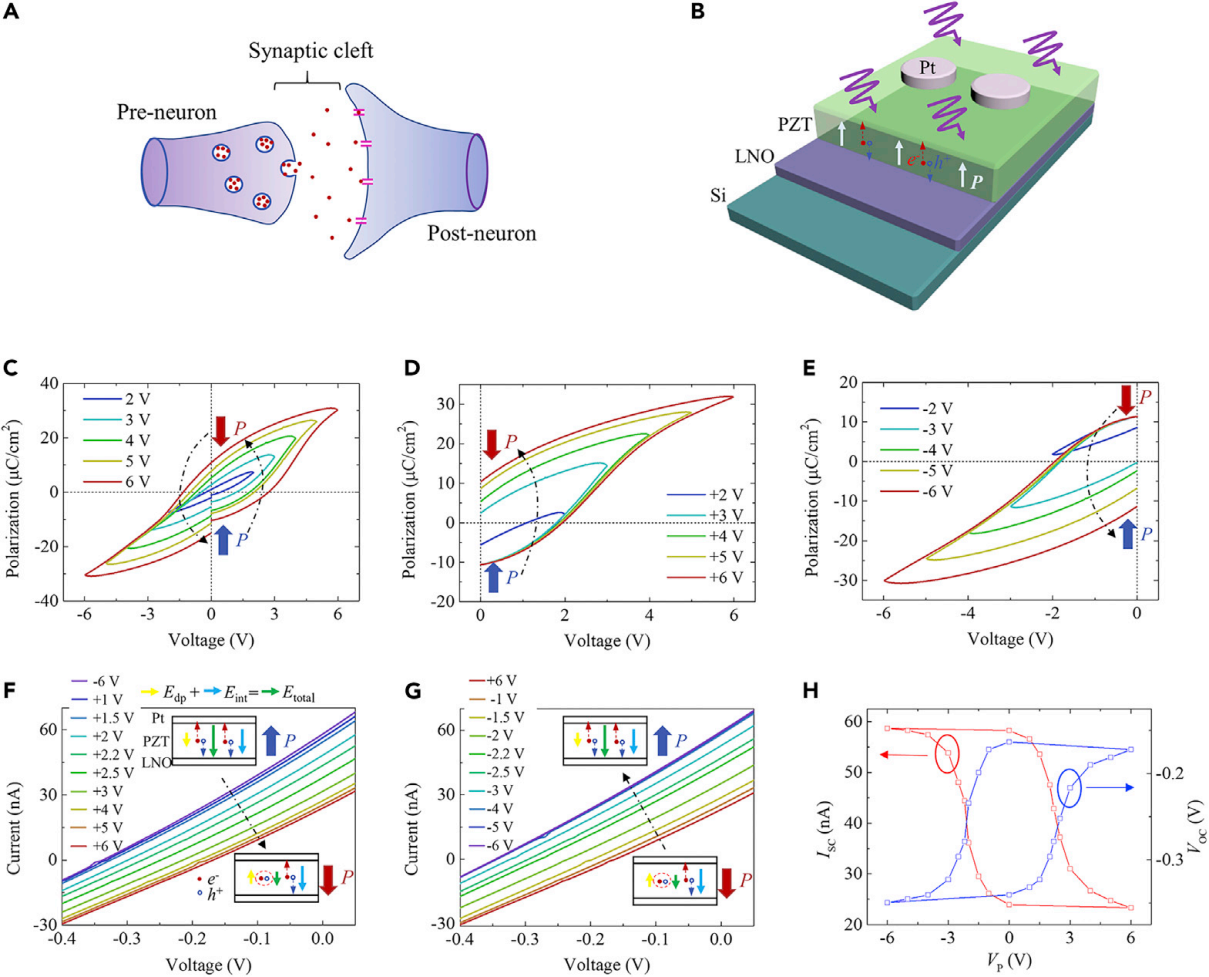
Polarization-Modulated Photovoltaic Response in the FePV Device (A and B) Schematic illustrations of the structures of (A) a biological synapse and (B) a Pt/PZT/LNO FePV device grown on a silicon substrate. (C–G) (C) Bipolar, (D) positive monopolar, and (E) negative monopolar *P-V* hysteresis loops measured with different applied voltages (frequency: 3.3 kHz). Illuminated *I-V* characteristics measured after applying (F) positive pulses from +1 V to +6 V (starting from the initial −6-V pulse-written state) and (G) negative pulses from −1 V to −6 V (starting from the initial +6-V pulse-written state). Insets in (F) and (G) show the variation of *E*_total_ (vector sum of *E*_dp_ and *E*_int_) during the polarization switching. (H) Evolutions of *I*_SC_ and *V*_OC_ as a function of pulse amplitude (*V*_p_).

The human brain can outperform the most advanced digital computer in many intellectual tasks, such as image and voice recognition, data classification, and associative learning (Drachman, 2005; Banerjee et al., 2017; Kuzum et al., 2013; Markram, 2012). Moreover, the human brain consumes only ∼20 W power to perform an intellectual task, which is at least three orders of magnitude lower than that of the digital computer (Eryilmaz et al., 2015; Yang et al., 2018b). Therefore, the brain-inspired neuromorphic computing is considered as a very promising computing architecture in the coming era of artificial intelligence. The learning in the brain is enabled by the ability of synapses (Figure 1A) to strengthen or weaken their connection strengths (or weights) in response to external stimuli, which is called synaptic plasticity (Abbott and Nelson, 2000). Artificial synaptic devices that can emulate the synaptic plasticity are, therefore, a key building block for a neuromorphic computing system. A simple yet energy-efficient synaptic device is the memristor, whose resistance can be continuously tuned depending on the history of electrical signals (Chang et al., 2011; Ohno et al., 2011). Most reported memristors are based on filament-forming oxides (Yan et al., 2018; Guan et al., 2019), electrolyte-gated oxides and polymers (Ge et al., 2019; Gkoupidenis et al., 2015), two-dimensional (2D) nitrides and sulfides (Shi et al., 2017; Wang et al., 2018; Li et al., 2018), and phase change materials (Tuma et al., 2016; Kuzum et al., 2012; Ge et al., 2020). These memristors operate through the migration and ordering of ions or atoms, which are inherently stochastic and difficult to control (Burr et al., 2018). Large device variability and poor reliability are therefore ubiquitous in these memristors.

A promising alternative to the aforementioned memristors is the ferroelectric synapse, whose synaptic behavior is induced by a purely electronic switching process, namely, polarization switching. There have been two types of ferroelectric synapses demonstrated so far: ferroelectric tunnel junctions (FTJs) (Chanthbouala et al., 2012; Wen et al., 2014; Li et al., 2019; Majumdar et al., 2019; Boyn et al., 2017; Guo et al., 2018; Chen et al., 2018; Ma et al., 2020) and ferroelectric field effect transistors (FeFETs) (Hoffman et al., 2010; Nishitani et al., 2012; Tian et al., 2019; Kim and Lee, 2019; Luo et al., 2020), which use the switchable polarization to tune the tunneling current and channel current, respectively. Although both FTJs and FeFETs have shown large ON/OFF ratios, low energy dissipations, and synaptic functions including short-/long-term plasticity and spike-timing-dependent plasticity (STDP) (Li et al., 2019; Majumdar et al., 2019; Boyn et al., 2017; Guo et al., 2018; Chen et al., 2018; Nishitani et al., 2012; Tian et al., 2019), they are still facing challenges in terms of the controllability of polarization and the device fabrication. Specifically, an FTJ requires the ferroelectric film to be ultra-thin (several nanometers) to allow the current tunneling, whereas an FeFET needs to use a semiconductor channel whose screening ability is apparently lower than that of a metal electrode. Both ultra-small film thickness (Kohlstedt et al., 2005; Cai et al., 2011) and poor screening at the ferroelectric/semiconductor interface (Wurfel et al., 1973) can lead to a large depolarization field (*E*_dp_). The large *E*_dp_ in turn causes the polarization instability, making it difficult to precisely control the polarization state and associated conductance level. In addition, an FTJ typically requires strained epitaxy of an ultra-thin ferroelectric film on a single-crystalline oxide substrate (Chanthbouala et al., 2012; Wen et al., 2014; Li et al., 2019; Majumdar et al., 2019; Boyn et al., 2017; Guo et al., 2018), whereas for an FeFET a careful optimization of the ferroelectric/semiconductor interface quality is demanded (Hoffman et al., 2010; Nishitani et al., 2012; Tian et al., 2019; Kim and Lee, 2019). The limited polarization controllability as well as complex and costly fabrication processes may, therefore, become the major challenges for both FTJs and FeFETs to be used as synaptic devices in hardware-based neural networks.

To address the above-mentioned challenges, ferroelectric synapses with a new way to read out the polarization state, other than the tunneling current in FTJs and the channel current in FeFETs, should be explored. Ferroelectric photovoltaic (FePV) effect offers a viable way of readout, i.e., polarization-controlled switchable photocurrent (Yuan et al., 2014; Fan et al., 2015; Ji et al., 2010; Yi et al., 2011; Guo et al., 2013). Using the photocurrent as the readout, ferroelectric materials (in any form and with a broad range of thicknesses) (Tan et al., 2018, 2019; Yang et al., 2010; Qin et al., 2009; Chen et al., 2011; Bai et al., 2018; He et al., 2019; Blouzon et al., 2016; Alexe and Hesse, 2011) sandwiched between two metal electrodes can in principle function as FePV synapses. Therefore, the limitations in FTJs (ultra-small film thickness and epitaxial growth) and FeFETs (semiconductor channel and optimization of interface quality) no longer exist in the FePV synapses. One consequence is that the *E*_dp_ effect can be reduced in the FePV synapses and hence the controllability of polarization is enhanced, beneficial for the performance of neural networks built from these devices. Another consequence is that the FePV synapses with simple structures can be fabricated using a wide variety of low-cost techniques and substrates. These advantages suggest the immense application potential of FePV synapses in neuromorphic computing architectures. It is, however, noted that whereas the FePV effect was once used for the binary data storage (Guo et al., 2013), the use of the FePV effect for synaptic applications has never been attempted yet.

Herein, we develop a proof-of-concept FePV synapse with a simple two-terminal structure of Pt/Pb(Zr_0.2_Ti_0.8_)O_3_ (PZT)/LaNiO_3_ (LNO) (Figure 1B). PZT with a Zr/Ti ratio of 20/80 is chosen as the FePV material because it possesses robust ferroelectricity, strong photoresponse in the UV wavelength region (Tan et al., 2019), and wide process window. The Pt/PZT/LNO FePV device can be facilely grown on a silicon substrate, showing its good compatibility with the silicon technology. More importantly, it exhibits gradual polarization switching behavior benefitting from the multi-domain switching with relatively slow dynamics in the polycrystalline PZT film. This gives rise to multilevel nonvolatile photovoltaic responses, which can be continuously tuned with small nonlinearity and write noise, highlighting the good controllability. Using the photovoltaic response as the synaptic weight, the FePV device exhibits various synaptic functions, such as long-term potentiation (LTP), long-term depression (LTD), and STDP. A FePV synapse-based neural network is further simulated, and it achieves high accuracies (>93%) for image recognition, comparable to those achieved by neural networks based on high-quality FTJs and FeFETs. These highly controllable and silicon-compatible FePV synapses are therefore a promising candidate for synaptic applications.

## Results and Discussion

### Gradual Polarization Switching in Polycrystalline PZT Film

The key component of the proposed FePV synapse is the polycrystalline PZT film (Figure S1) exhibiting gradual polarization switching, which enables the access to multilevel photovoltaic responses. The polarization switching behavior was first investigated by measuring bipolar and monopolar polarization-voltage (*P-V*) hysteresis loops. Figure 1C shows that the bipolar *P-V* loop opens gradually as the amplitude of applied voltage increases. The remanent polarization (*P*_r_) can reach ∼10 μC/cm^2^, consistent with that reported for polycrystalline PZT (Meng et al., 2000). In addition, these bipolar *P-V* loops have a slanted shape, indicative of gradual polarization switching. The monopolar *P-V* loops, shown in Figures 1D and 1E, further illustrate that the polarization is gradually switched to the downward (upward) direction as the amplitude of applied positive (negative) voltage increases.

### Gradual Polarization Switching-Induced Multilevel Photovoltaic Responses

To investigate the effect of gradual polarization switching on the photovoltaic response, current-voltage (*I-V*) characteristics under illumination were measured for the FePV device in different polarization states. UV light with a wavelength of 365 nm and an intensity of 105 mW/cm^2^ was used for illumination (Figure S2). The polarization was first set in the fully upward (downward) state by applying a −6 V (+6 V) write pulse with a width of 1 ms. Then, a series of positive (negative) write pulses with increasing amplitudes (pulse width: 10 μs) was applied sequentially to produce intermediate polarization states. No erase pulses were applied between these write pulses. In each intermediate polarization state, the illuminated *I-V* characteristics were measured with a sufficiently low voltage sweeping speed (Figure S3A). The dark *I-V* characteristics were also measured, and the dark currents at low voltages were found to be orders of magnitude smaller than the photocurrent (Figure S3B). The applied voltage is defined to be positive when the top electrode is positively biased, and the current is termed positive when it flows from top to bottom.

As shown in Figure 1F, the FePV device exhibits noticeable photovoltaic behavior with a short-circuit current (*I*_SC_) of ∼57 nA and an open-circuit voltage (*V*_OC_) of ∼ -0.33 V in the fully upward polarization state (i.e., the −6-V pulse-written state). Starting from this state, applying positive write pulses from +1 V to +6 V leads to the successive shift of the illuminated *I-V* curve toward the origin, indicating the continuous decrease of photovoltaic response. Eventually, *I*_SC_ and *V*_OC_ decrease to ∼23 nA and ∼-0.15 V, respectively, in the fully downward polarization state (i.e., the +6-V pulse-written state). All the available photovoltaic responses are rather stable against time (Figure S4), attesting to the nonvolatility of the emerging intermediate photoresponsive states. On the contrary, applying negative write pulses from −1 V to −6 V leads to the successive shift of the illuminated *I-V* curve away from the origin (Figure 1G). Moreover, the values of *I*_SC_ and *V*_OC_ are almost recovered to those in the initial −6-V pulse-written state (see comparison between Figures 1F and 1G). Figure 1H presents the evolutions of *I*_SC_ and *V*_OC_ as a function of write pulse amplitude, both of which form well-shaped hysteresis loops akin to the *P-V* hysteresis loops. Similar successive shift of the illuminated *I-V* curve and hysteretic evolutions of *I*_SC_ and *V*_OC_ with applied write pulse are observed at different light intensities (Figures S5 and S6). Apparently, the observed reversible multilevel modulation of photovoltaic response is associated with the gradual polarization switching in the polycrystalline PZT film (Figures 1C–1E). Notably, if the polarization switching is abrupt, only two bistable photoresponsive states can commonly be accessed, as observed in epitaxial ferroelectric films (Figure S7).

The modulation of photovoltaic response by polarization is further confirmed by microscopic observations of concurrent changes in domain configuration and photocurrent (Figures S8 and S9). The mechanism of how the polarization controls the photovoltaic response in our FePV device is discussed in detail with Figure S10. Briefly, a switchable *E*_dp_ and an unswitchable internal bias field (*E*_int_) may be considered as the driving forces for the photovoltaic effect (Ji et al., 2010; Fan et al., 2017a). In the fully upward polarization state, both *E*_dp_ and *E*_int_ are oriented downward, resulting in a large total field (*E*_total_) pointing downward (inset in Figure 1F). When the polarization is switched downward, *E*_dp_ rotates to the upward direction, whereas *E*_int_ remains unchanged (inset in Figure 1F). Given that |*E*_int_| is larger than |*E*_dp_|, the resultant *E*_total_ is still pointing downward but its magnitude is smaller than that in the fully upward polarization state. As a result, the magnitudes of *I*_SC_ and *V*_OC_ decrease (increase), whereas their signs remain unchanged as the polarization switches from upward to downward (downward to upward) (Figures 1F and 1G). As the gradual polarization switching induces a dynamic modulation of *E*_total_, multilevel photovoltaic responses are thus produced.

### Domain Dynamics Underlying Gradual Polarization Switching

**Figure 2:**
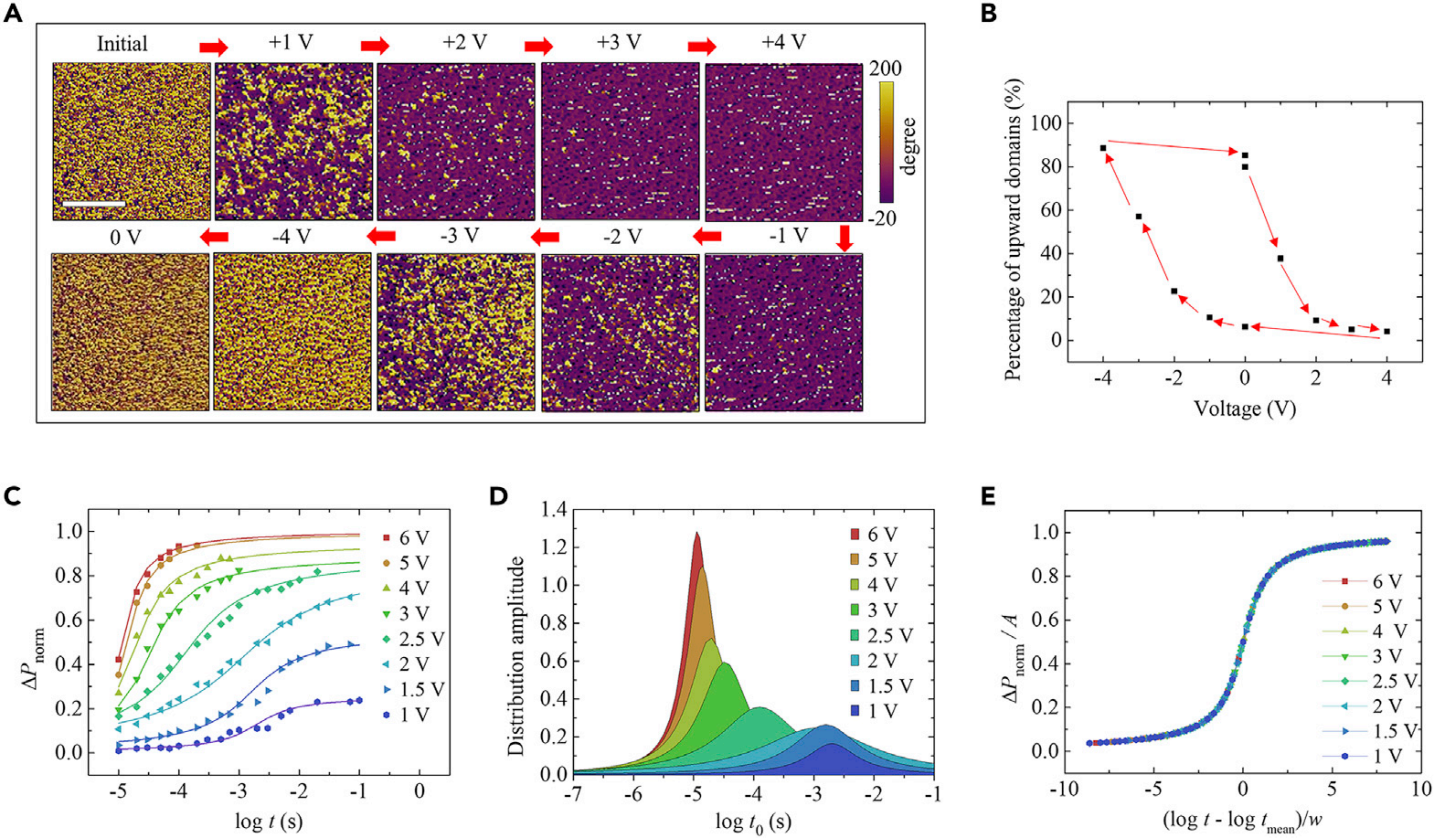
Domain Switching Kinetics in the Polycrystalline PZT Film (A) PFM phase images measured on the bare PZT film after applying different write voltages (varying as 0 → +4 V → −4 V → 0). Scale bar, 1 μm. (B) Area percentage of upward domains as a function of write voltage, as statistically obtained from (A). (C) Normalized switchable polarization (Δ*P*_norm_) versus pulse width under different pulse amplitudes measured using the PUND method. (D) Lorentzian distributions of characteristic switching times (*t*_0_) extracted from the fits in (C). (E) Rescaled Δ*P*_norm_ (Δ*P*_norm_/*A*) as a function of pulse width using the parameters for the NLS model.

Because the gradual polarization switching is crucial for realizing continuously tunable photovoltaic response, further understanding of the underlying domain dynamics is required. Figure 2A shows the evolution of domain configuration in the polycrystalline PZT film with varying write voltage, measured by piezoresponse force microscopy (PFM). As the write voltage increases from +1 V to +4 V, the purple areas with downward domains gradually expand, whereas the yellow areas with upward domains gradually shrink. Opposite domain evolution is observed as the write voltage varies from −1 V to −4 V. Interestingly, the plot of area percentage of upward domains against write voltage forms a hysteresis loop (Figure 2B), akin to the *P-V* hysteresis loops (Figure 1C). These PFM imaging results therefore directly reveal the continuous domain evolution behavior at the microscopic level. The continuous domain evolution is attributed to the fact that the polycrystalline PZT film possesses multi-domains with small sizes (minimum size of an individual domain is below ∼100 nm) facilitating the formation of multiple nonvolatile domain configurations during the domain switching.

To gain deeper insights into the domain dynamics, switchable polarizations (Δ*P*) as a function of pulse amplitude and width were measured using a positive-up-negative-down (PUND) method (Schloss and McIntyre, 2003; Borkar et al., 2017). In the PUND method, the first and second pulses with the same polarity measure the total polarization (*P*_total_) and nonswitchable polarization (*P*_ns_), respectively. Δ*P* is obtained by subtracting *P*_ns_ from *P*_total_. Figure 2C displays that the normalized Δ*P* (Δ*P*_norm_) systematically increases with increasing pulse amplitude at a given pulse width or with increasing pulse width at a given pulse amplitude. This indicates that the domains take a longer (shorter) time to be switched at a lower (higher) electric field. To quantitatively describe the switching kinetics, the nucleation-limited switching (NLS) model (Jo et al., 2007; Tagantsev et al., 2002) was employed. In this model, domain switching occurs region by region independently, and the switching in each region is governed by the nucleation of reversed domain. The time-dependent Δ*P* can be expressed as:

(Equation 1) $$\Delta P\left(t\right)=2P_{s}\int_{-\infty }^{\infty }\left[1-\exp\left\{-{\left(t/t_{0}\right)}^{n}\right\}\right]F\left(\log\;t_{0}\right)d\left(\log\;t_{0}\right)$$

where *P*_s_ is the spontaneous polarization (Δ*P*/2*P*_s_ is indeed Δ*P*_norm_), *t*_0_ is the characteristic switching time, *n* is the effective dimension (*n* = 2 for thin films, Jo et al., 2007), and *F*(log*t*_0_) is Lorentzian distribution of the logarithm of switching times. The expression of *F*(log*t*_0_) is given by:

(Equation 2) $$F\left(\log\;t_{0}\right)=\frac{A}{\pi }\left[\frac{w}{{\left(\log\;t_{0}-\log\;t_{\text{mean}}\right)}^{2}+w^{2}}\right]$$

where *A* is an amplitude factor, *t*_mean_ is the mean switching time (i.e., the center of the distribution curve peak), and *w* is the half-width at half-maximum of the distribution curve peak.

As shown in Figure 2C, the experimental data of Δ*P*_norm_(*t*) can be well fitted by the NLS model. Figure 2D presents the Lorentzian distribution curves at different pulse amplitudes. As the pulse amplitude increases, the distribution curve peak shifts leftward and becomes sharper, corresponding to the decreases of *t*_mean_ and *w*, respectively. *t*_mean_ decreases from ∼10^−3^ to ∼10^−5^ s as pulse amplitude increases from 1 to 6 V (corresponding to 50 to 300 kV/cm). These *t*_mean_ values are at least one order of magnitude larger than those in epitaxial ferroelectric films in the same electric field range (So et al., 2005), well accounting for the gradual feature of polarization switching in our polycrystalline PZT film.

By rescaling the Δ*P*_norm_(*t*) data using (log*t* − log*t*_mean_)/*w*, all the curves merge into a single arctangent curve (Figure 2E). This scaling behavior suggests that the switching times obey the Lorentzian distribution. Moreover, the dependence of the mean switching time *t*_mean_ on the reciprocal of electric field follows the Merz's law (Jo et al., 2007) (Figure S11). These results in turn validate the NLS model. Therefore, the nucleation-limited multi-domain switching with relatively slow dynamics gives rise to the gradual polarization switching in our polycrystalline PZT film.

### Modulation of Photovoltaic Response by Electrical Pulse

With the aforementioned understandings, the gradual polarization switching-induced multilevel photovoltaic responses in the FePV device were further characterized. The photocurrent density at 0 V per unit light intensity, denoted by *J*_ph_^∗^ with the superscript “∗” meaning “per 1 mW/cm^2^,” was used as a quantitative term to represent the photovoltaic response. Note that the *J*_ph_^∗^ data presented hereafter were measured at a light intensity of 105 mW/cm^2^ and those measured at other light intensities are shown in Figures S12 and S13.

**Figure 3:**
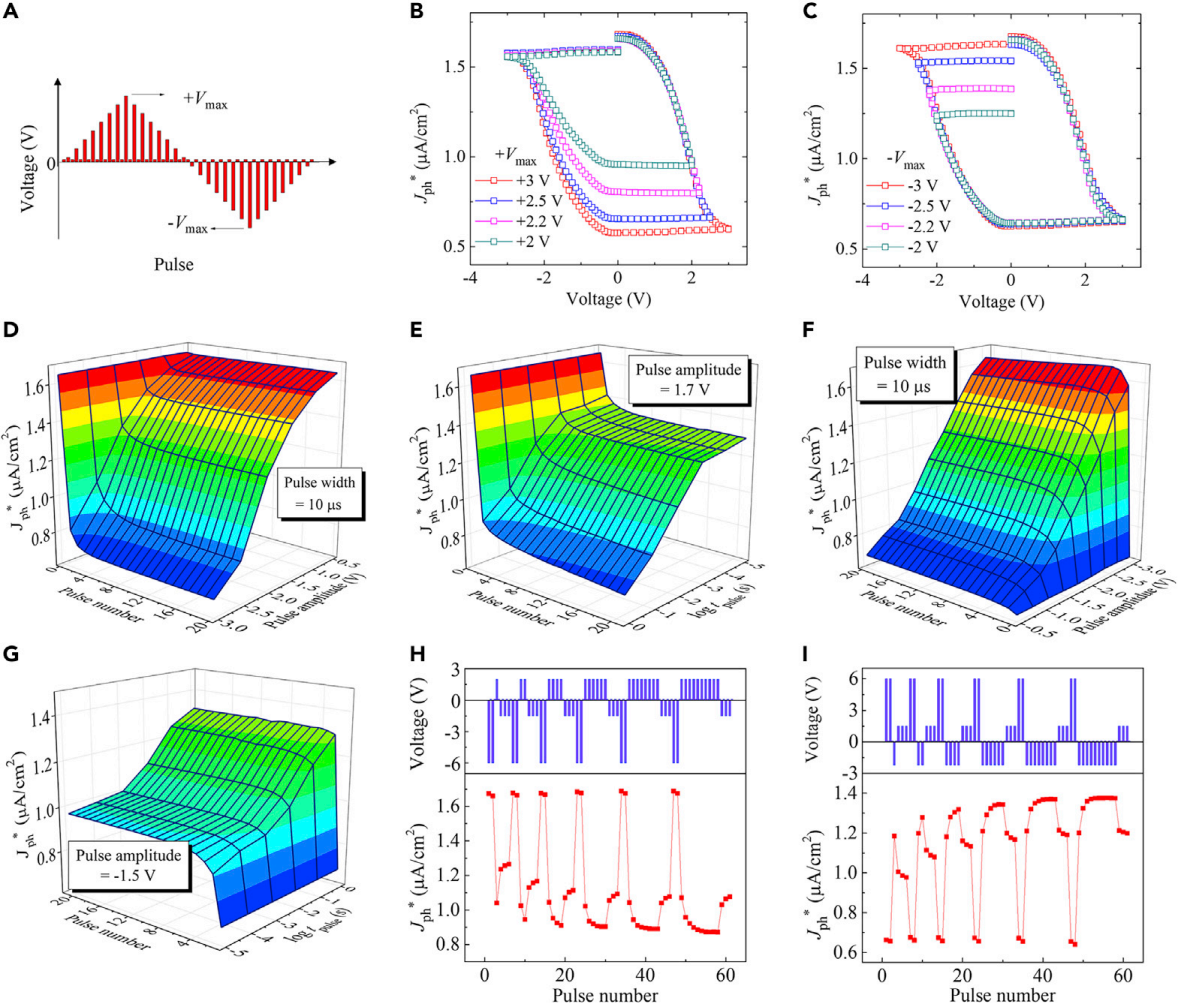
Continuously Tunable Photovoltaic Response (*J*_ph_^∗^) in the FePV Synapse (A–G) (A) A schematic showing the pulse train following a triangular profile. Dependences of *J*_ph_^∗^ on the pulse amplitude measured using the pulse trains (shown in A) with varying (B) +*V*_max_ and (C) -*V*_max_. Evolutions of *J*_ph_^∗^ measured using repeated pulses with (D and F) varying pulse number and pulse amplitude while fixing the pulse width and (E and G) varying pulse number and pulse width while fixing the pulse amplitude. (H) Evolution of *J*_ph_^∗^ (lower panel) measured using the negative-positive-negative pulse train (upper panel) where the number of positive pulses (+2.2 V) between two negative pulse groups is varied. (I) Evolution of *J*_ph_^∗^ (lower panel) measured using the positive-negative-positive pulse train (upper panel) where the number of negative pulses (−2.2 V) between two positive pulse groups is varied.

Figures 3B and 3C show the hysteretic evolutions of *J*_ph_^∗^ as the pulse amplitude varies following triangular profiles (see Figure 3A) while keeping the pulse width at 1 ms. Starting from the same state written by the −6-V pulse, applications of positive pulse trains with maximum amplitudes of +2, +2.2, +2.5, and +3 V result in intermediate *J*_ph_^∗^ levels of ∼0.95, ∼0.80, ∼0.66, and ∼0.60 μA/cm^2^, respectively (Figure 3B). Likewise, intermediate *J*_ph_^∗^ levels ranging from ∼1.24 to ∼1.38, ∼1.54, and ∼1.62 μA/cm^2^ can be obtained by applying negative pulse trains with maximum amplitudes increasing from −2 to −2.2, −2.5, and −3 V, respectively. All these intermediate *J*_ph_^∗^ levels are nonvolatile based on the observations of almost flat bottom and top parts of the hysteresis loops (Figures 3B and 3C).

Besides the triangular pulse trains, repeated pulses were also used to tune the polarization states and consequent *J*_ph_^∗^ levels. As shown in Figures 3D and 3E, the initially set high *J*_ph_^∗^ decreases and eventually reaches a saturated value as the number of applied positive pulses increases. The saturation of *J*_ph_^∗^ can be explained by the fact that the domains that are responsive under this pulse amplitude and width are switched during the first few pulses and then no more domains can be switched with further increasing pulse number. In addition, the variation of *J*_ph_^∗^ from the initial value to the saturated value becomes larger with larger pulse amplitude and longer pulse duration, due to the switching of more domains. Similar dependences of the *J*_ph_^∗^ variation on pulse amplitude and width are observed when tuning the initial low-*J*_ph_^∗^ state by applying repeated negative pulses (Figures 3F and 3G).

Then, the effects of the history of applied pulses on *J*_ph_^∗^ were investigated by applying consecutive pulse trains containing alternate positive and negative pulses. Figure 3H (upper panel) shows the sequence of a negative-positive-negative pulse train: two −6-V pulses (for setting the high-*J*_ph_^∗^ state), +2.2-V pulses with varied numbers, and three −1.5-V pulses (the widths of all pulses are 1 ms). Applying this pulse train to the FePV device produces the evolution of *J*_ph_^∗^ shown in Figure 3H (lower panel). Interestingly, the *J*_ph_^∗^ value after each group of −1.5-V pulses gradually decreases, which can be attributed to the increasing number of previously applied +2.2-V pulses. Similar history dependence of the *J*_ph_^∗^ variation is also observed when applying a positive-negative-positive pulse train (Figure 3I).

### Synaptic Functions of the FePV Synapse

**Figure 4:**
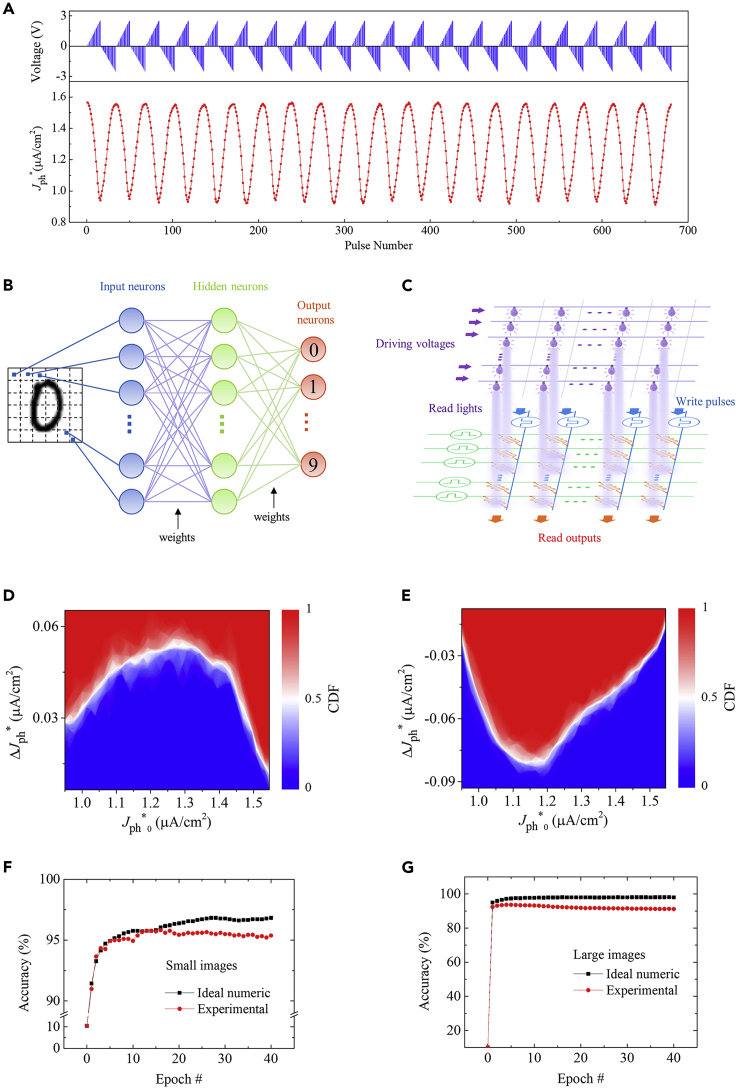
Long-Term Plasticity and Neural Network Simulation (A) LTD and LTP characteristics (lower panel) measured using the alternate positive and negative pulse trains (upper panel). (B) A schematic showing a three-layer (one hidden layer) neural network. (C–G) (C) Schematics of a crossbar architecture based on the FePV synapses and the light arrays used for the read operation. Probability distributions of the change in *J*_ph_^∗^ induced by a write operation (i.e., Δ*J*_ph_^∗^) versus initial *J*_ph_^∗^ (i.e., *J*_ph_^∗^_0_) for (D) potentiation and (E) depression. CDF denotes the cumulative distribution function. Evolutions of accuracies with training epochs achieved by the ideal floating-point-based and the FePV synapse-based neural networks for recognizing (F) small (8 × 8 pixels) and (G) large (28 × 28 pixels) images.

Because the FePV device exhibits continuous modulation of *J*_ph_^∗^ depending on the amplitude, duration, and history of applied pulses, it can thus be qualified as a synaptic device with the tunable photovoltaic response (i.e., *J*_ph_^∗^) corresponding to the synaptic weight. To further demonstrate the synaptic plasticity of the FePV device, the STDP behavior was characterized, as shown in Figure S14. In addition, LTP and LTD characteristics were also measured using an increasing-voltage pulse scheme (upper panel of Figure 4A). In this pulse scheme, a positive pulse train (amplitude: from 0 to +2.5 V in increments of ∼0.17 V; width: 10 μs) and a negative pulse train (amplitude: from 0 to −2.5 V in decrements of ∼ -0.17 V; width: 10 μs) were applied alternately, and *J*_ph_^∗^ was measured after each pulse. The reason for using this pulse scheme was because more domains become responsive and can be switched under larger pulse amplitude, thus allowing a continuously tuned photocurrent with increasing pulse number.

As shown in Figure 4A, *J*_ph_^∗^ gradually decreases as the number of positive pulses increases, indicating an LTD behavior. Conversely, the LTP behavior, manifesting as the increase of *J*_ph_^∗^, occurs during the application of the negative pulse train. These results are consistent with those in Figures 1 and 3, all of which can be explained by the polarization modulation of photovoltaic response. Almost identical LTD and LTP processes can be repeated for 20 cycles, demonstrating a small cycle-to-cycle variation (∼2%). In addition, there are 16 different *J*_ph_^∗^ levels in the LTD and LTP processes, confirming that multiple photoresponsive states are accessible.

### FePV Synapse-Based Neural Network

Using the experimentally measured cyclic LTP/LTD characteristics to map the synaptic weights, we further simulated a FePV synapse-based neural network for image recognition (Figures 4B and 4C). A three-layer (one hidden layer) neural network, as shown in Figure 4B, was used for the back-propagation algorithm-based simulations (Saerens and Soquet, 1991; Hegazy et al., 1994). Each synaptic weight matrix between two neuron layers was modeled as a crossbar (Figure 4C). The crossbar performed vector-matrix multiply and parallel rank one outer product update, with our FePV devices acting as the synapses in the crossbar. The neural network was trained and tested on two datasets: a small image version (8 × 8 pixels) of handwritten digits from the “Optical Recognition of Handwritten Digits” dataset (Kang and P.-Brown, 2008) and a large image version (28 × 28 pixels) of handwritten digits from the “Modified National Institute of Standards and Technology” (MNIST) dataset (Deng, 2012). The pixel values of the images were encoded as the light intensities used to illuminate the FePV synapses (see Figure S15 for detailed descriptions about the operations of the FePV synapse-based neural network).

Generally, the performance of a neural network is greatly influenced by the controllability (e.g., nonlinearity and write noise) of synaptic devices, which can be quantitatively analyzed using the probability distribution of the change in *J*_ph_^∗^ induced by a write operation (i.e., Δ*J*_ph_^∗^). The plots of Δ*J*_ph_^∗^ versus initial *J*_ph_^∗^ (i.e., *J*_ph_^∗^_0_), derived from the cyclic LTP/LTD characteristics, are presented in Figures 4D and 4E for potentiation and depression, respectively. The magnitude of Δ*J*_ph_^∗^ first increases and then decreases with increasing (decreasing) *J*_ph_^∗^_0_ in the potentiation (depression) process. The average slopes of Δ*J*_ph_^∗^ versus *J*_ph_^∗^_0_ are ∼0.12 and ∼0.21 for potentiation and depression, respectively, which are relatively small and thus demonstrate the small nonlinearity of our FePV synapses (note that a slope of zero means that the synaptic device is ideally linear). In addition, Δ*J*_ph_^∗^ deviates with an average standard deviation (σ_S_) of 4.75 × 10^−3^ μA/cm^2^ (4.96 × 10^−3^ μA/cm^2^) for potentiation (depression). The average signal-to-noise ratios Δ*J*_ph_^∗2^/σ_S_^2^ are ∼80 and ∼127 for potentiation and depression, respectively, which are quite large and comparable to those observed in the state-of-the-art Li-ion synaptic transistors (Fuller et al., 2017). The small nonlinearity and write noise of our FePV synapses may render good performance for the neural network.

Figures 4F and 4G show the recognition accuracies of the FePV synapse-based neural network after training with small and large images, respectively. The results of the ideal floating-point-based neural network are also shown, which represent the theoretical limits for the neuromorphic algorithm. For small images, the accuracy exceeds 90% after the first two training epochs and approaches ∼95.4% after 40 training epochs, which is only ∼1.4% lower than the ideal accuracy (∼96.8%). For large images, the accuracy reaches a maximum value of ∼93.7% after 4 training epochs, which deviates the ideal accuracy (∼98.0%) by ∼4.3%. Further increasing the training epoch leads to the decrease of accuracy, probably due to the issue of overfitting. Nevertheless, these accuracies rank high among those obtained with FTJs (Li et al., 2019; Ma et al., 2020), FeFETs (Yang et al., 2018a; Kim and Lee, 2019), filament-forming oxides (Choi et al., 2018), and phase change materials (Ge et al., 2019, 2020) (see Table S1 for details), demonstrating the great potential of FePV synapses for application in high-performance neuromorphic computing. Note that in the above simulations the simulated accuracy already took into account the effect of the cycle-to-cycle variation because the cyclic LTP/LTD characteristics were used. The effects of other non-ideal factors of the FePV synapses on the simulated accuracy can be found in Figure S16.

### Reliability and Energy Consumption of the FePV Synapse

**Figure 5:**
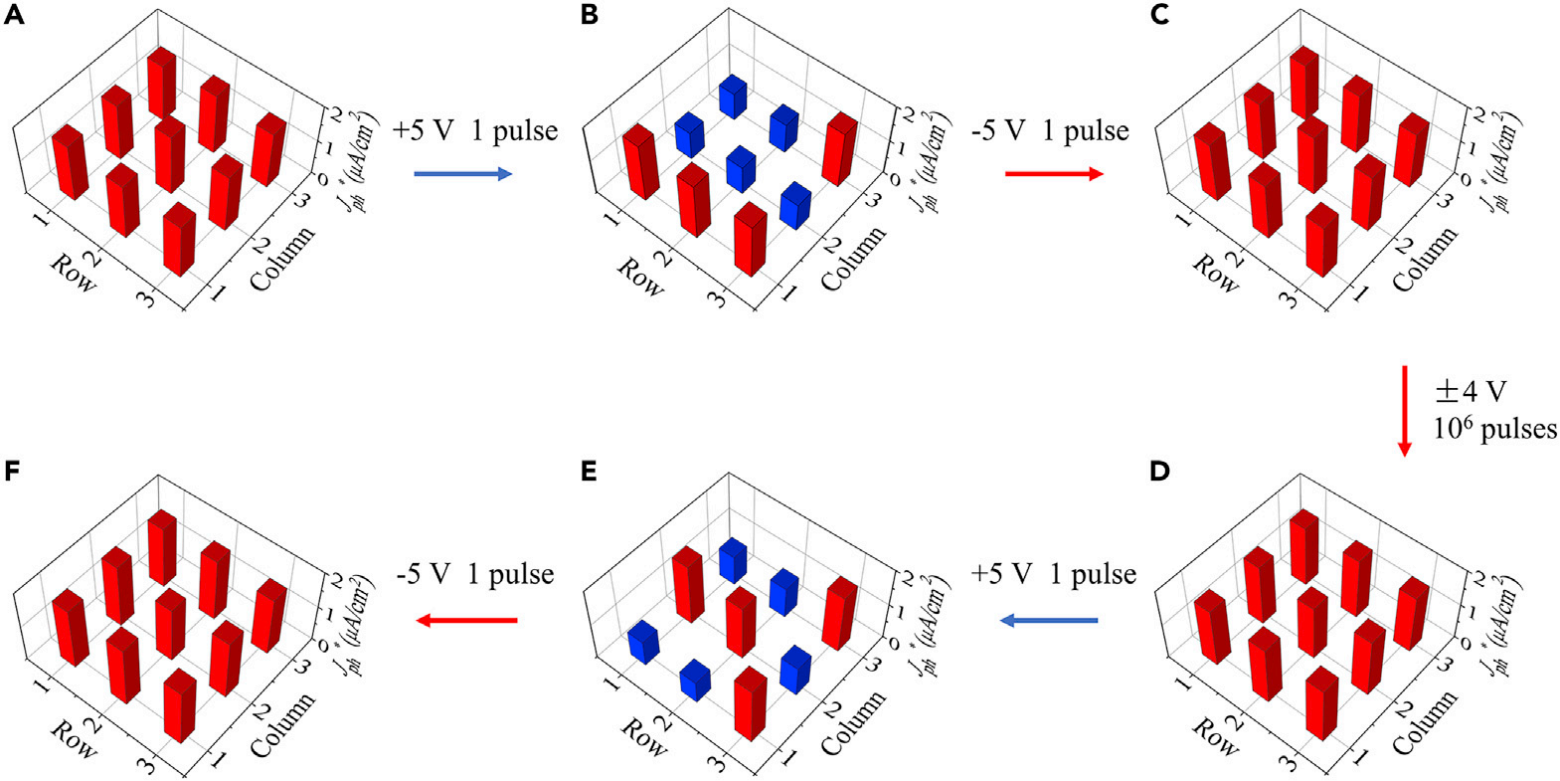
Device-to-Device Variation and Endurance (A) A 3 × 3 FePV synapse array with uniformly high *J*_ph_^∗^ values in the initial state. (B and C) Distribution of *J*_ph_^∗^ values after applying (B) +5-V and (C) −5-V pulses to each of the five selected FePV synapses (forming a letter “P”). (D) The cyclic switching of all FePV synapses by applying ±4-V pulses repeatedly for 10^6^ cycles. (E and F) Distribution of *J*_ph_^∗^ values after applying (E) +5-V and (F) −5-V pulses to each of other five selected FePV synapses (forming a letter “V”).

For a neural network, besides the accuracy, the reliability is also a key concern. While the cycle-to-cycle variation and retention of the constituent synaptic devices have already been addressed, the device-to-device variation and endurance are yet to be measured. Measurements on the device-to-device variation and endurance were performed with an array of 3 × 3 separated FePV synapses, as shown in Figure 5. In the initial state (Figure 5A), the *J*_ph_^∗^ values of all FePV synapses are distributed uniformly at ∼1.65 μA/cm^2^. After applying a +5-V pulse (10 μs) to each of five selected FePV synapses (forming a letter “P”), the *J*_ph_^∗^ values of these devices decrease uniformly to ∼0.81 μA/cm^2^ (Figure 5B). The *J*_ph_^∗^ values can be recovered to ∼1.65 μA/cm^2^ by simply applying a −5-V pulse (10 μs) to each of the selected FePV synapses (Figure 5C). All the FePV synapses were then switched by ±4-V pulses repeatedly for 10^6^ cycles (note: the pulse amplitude was selected to be ∼1.5 times the coercive voltage, following the standard protocol used for the endurance test). Afterward, the pulse modulations of the *J*_ph_^∗^ values of other five selected FePV synapses (forming a letter “V”) were demonstrated, and the *J*_ph_^∗^ variations were observed to be similar to those before the endurance test, as shown in Figures 5D–5F. Detailed evolutions of polarization and *J*_ph_^∗^ with the number of switching cycles are presented in Figure S17, revealing that both polarization and *J*_ph_^∗^ change slightly even after switching for 10^6^ cycles. These results demonstrate that our FePV synapses have small device-to-device variation and good endurance.

The last concern is the energy consumption. For the FePV synapse presented in this work, the write energy is calculated to be on the order of ∼10 nJ (Figure S18), which is much higher than the fJ level as reported previously (Ma et al., 2020; Tian et al., 2019). The reasons for the high write energy include the large device area (3.14 × 10^−4^ cm^2^) and large pulse width (≥10 μs). Indeed, we previously demonstrated that the switchable photovoltaic response could be observed in an FePV device as small as ∼1 μm^2^ (Fan et al., 2017b). In addition, as indicated by Figure 2C, to switch the same amount of polarization, a small increase in the pulse amplitude can lead to a decrease of the pulse width by orders of magnitude. For example, a 12.3-V pulse can switch a polarization of ∼134 μC/cm^2^ within a switching time of 1.7 ns, and the triggered current density was ∼9 × 10^4^ A/cm^2^ (Grigoriev et al., 2011). For our PZT film with a switchable polarization of ∼20 μC/cm^2^, the current density triggered by the 12.3-V/1.7-ns pulse may thus be ∼1.3 × 10^4^ A/cm^2^. Therefore, the write energy of our FePV synapse may be reduced to ∼2.7 pJ (12.3 V × 1.3 × 10^4^ A/cm^2^ × 1 μm^2^ × 1.7 ns). In terms of the read energy, only the light energy is considered because the photocurrent exists at zero read voltage. Using the light intensity of 105 mW/cm^2^ and the device area of 3.14 × 10^−4^ cm^2^, the light power is calculated as ∼0.03 mW. Although in this work the pulsed light was not used, it was reported that the FePV effect could occur by applying light pulses as short as ∼1 ns (Xing et al., 2015; Yang et al., 2020; Li et al., 2017; Gerasimenko et al., 2019). Also considering the device downscaling (assuming a device area of ∼1 μm^2^), it is therefore possible to bring the read energy of the FePV synapse to ∼1 aJ (105 mW/cm^2^ × 1 μm^2^ × 1 ns).

### Merits of the FePV Synapse

Finally, let us summarize the merits of the FePV devices as artificial synapses. Unlike the traditional memristive devices, which are defect-mediated, the FePV devices use the polarization to modulate the photovoltaic response, thus providing a more precise control over the synaptic weight. Compared with other ferroelectric synapses, like FTJs and FeFETs, the FePV synapses have two major advantages as follows. First, the FePV synapses have simple structures and facile fabrication procedures. Taking our FePV synapse as an example, it consists of a simple two-terminal Pt/PZT (polycrystalline film)/LNO structure, which can be easily grown on a silicon substrate, demonstrating its good compatibility with the silicon technology. More generally, ferroelectric materials in any form (thin films, Tan et al., 2018; Yang et al., 2010; Tan et al., 2019; Qin et al., 2009; Chen et al., 2011; ceramics, Bai et al., 2018; He et al., 2019; single crystals, Blouzon et al., 2016; Alexe et al., 2011; etc.) can be used for constructing FePV synapses because of the universality of the FePV effect. By contrast, FTJs and FeFETs typically require the epitaxy growth (Chen et al., 2018; Ma et al., 2020) and careful optimization of the ferroelectric/semiconductor interface quality (Hoffman et al., 2010), respectively, and some of them are difficult to be integrated with the silicon substrate. The second advantage of the FePV synapses is the ability to directly measure and precisely control the polarization. As a result, the coupling between polarization and photovoltaic response can be unambiguously demonstrated, which not only makes clear the mechanism of the polarization-mediated synaptic behavior but also allows us to better control the synaptic weight. However, the direct measurement and precise control of polarization are still challenging for both FTJs (Boyn et al., 2017) and FeFETs (Tian et al., 2019), because of the ultra-thin ferroelectric films and semiconductor channels used in FTJs and FeFETs, respectively.

## Conclusions

To sum up, we have proposed and demonstrated a prototype FePV synapse based on a polycrystalline PZT film sandwiched between Pt and LNO electrodes grown on a silicon substrate. This device relies on (1) the gradual polarization switching benefitting from the multi-domain switching with relatively slow dynamics and (2) the polarization control of photovoltaic effect, to achieve multilevel nonvolatile photovoltaic responses, as demonstrated by the combined *P-V* loop and PFM measurements. Moreover, the photovoltaic response (i.e., *J*_ph_^∗^) can be continuously and reversibly tuned by varying the amplitude, duration, and history of applied pulses, thus qualifying the FePV device as a synaptic device. Typical synaptic functions including LTP, LTD, and STDP have all been realized by the FePV synapses. More importantly, the FePV synapses also exhibit good controllability (small nonlinearity and write noise), high endurance, small device-to-device variation, and potentially low energy consumption. Consequently, a simulated neural network built from these devices achieves a high accuracy of ∼93.7% for recognizing the MNIST handwritten digits. Considering the above good performance as well as their simple fabrication and silicon compatibility, the FePV synapses may therefore represent a new type of hardware implementation of ferroelectric synapses for high-performance neuromorphic computing.

### Limitations of the Study

The photocurrent of the present FePV device is at the 10-nA level, which can be further enhanced if a ferroelectric material with a narrower band gap is used. A quantitative relationship between the photocurrent response and the pulse parameters, which can guide us to design the pulse scheme, is yet to be established. Although the FePV synapse-based neural network has been simulated and good performance has been predicted, the hardware implementation is still challenging. This is mainly because the uses of increasing-voltage pulse scheme for small nonlinearity and light signals (John et al., 2020) for reading would increase the circuit complexity. Encoding the image pixel values as the light intensities increases the number of operation events.

### Resource Availability

#### Lead Contact

Further information and requests for resources and reagents should be directed to and will be fulfilled by the Lead Contact, Prof. Zhen Fan (fanzhen@m.scnu.edu.cn).

#### Materials Availability

This study did not generate new unique materials.

#### Data and Code Availability

All data associated with the study are included in the paper and the Supplemental Information. Additional information is available from the Lead Contact upon reasonable request.

## Methods

All methods can be found in the accompanying Transparent Methods supplemental file.

## Supplemental Information

Document S1. Transparent Methods, Figures S1–S18, and Table S1

## Footnotes

Supplemental Information can be found online at https://doi.org/10.1016/j.isci.2020.101874.
