# Supplementary material for: Highly Controllable and Silicon-Compatible Ferroelectric Photovoltaic Synapses for Neuromorphic Computing
Source: iScience. 2020 Nov 30;23(12):101874. doi: 10.1016/j.isci.2020.101874 (PMC7736912; doi:10.1016/j.isci.2020.101874)
Supplement: Document S1. Transparent Methods, Figures S1–S18, and Table S1 [file mmc1.pdf]

iScience, Volume *23*

Supplemental Information

Highly Controllable and Silicon-Compatible

Ferroelectric Photovoltaic Synapses

for Neuromorphic Computing

Shengliang Cheng, Zhen Fan, Jingjing Rao, Lanqing Hong, Qicheng Huang, Ruiqiang Tao, Zhipeng Hou, Minghui Qin, Min Zeng, Xubing Lu, Guofu Zhou, Guoliang Yuan, Xingsen Gao, and Jun-Ming Liu

Supplemental Information

Highly-controllable and silicon-compatible ferroelectric photovoltaic synapses for neuromorphic computing

Shengliang Cheng[1,2], Zhen Fan[1,2,7*], Jingjing Rao[1], Lanqing Hong[3], Qicheng Huang[1], Ruiqiang Tao[1], Zhipeng Hou[1], Minghui Qin[1], Min Zeng[1], Xubing Lu[1], Guofu Zhou[2,4], Guoliang Yuan[5], Xingsen Gao[1] and Jun-Ming Liu[1,6]

[1]Institute for Advanced Materials, South China Academy of Advanced Optoelectronics, South China Normal University, Guangzhou 510006, China
[2]Guangdong Provincial Key Laboratory of Optical Information Materials and Technology, South China Academy of Advanced Optoelectronics, South China Normal University, Guangzhou 510006, China
[3]Department of Industrial Systems Engineering and Management, National University of Singapore, Singapore 117576, Singapore
[4]National Center for International Research on Green Optoelectronics, South China Normal University, Guangzhou 510006, China
[5]School of Materials Science and Engineering, Nanjing University of Science and Technology, Nanjing 210094, China
[6]Laboratory of Solid State Microstructures and Innovation Center of Advanced Microstructures, Nanjing University, Nanjing 210093, China
[7]lead contact
*Corresponding author. Email address: fanzhen@m.scnu.edu.cn

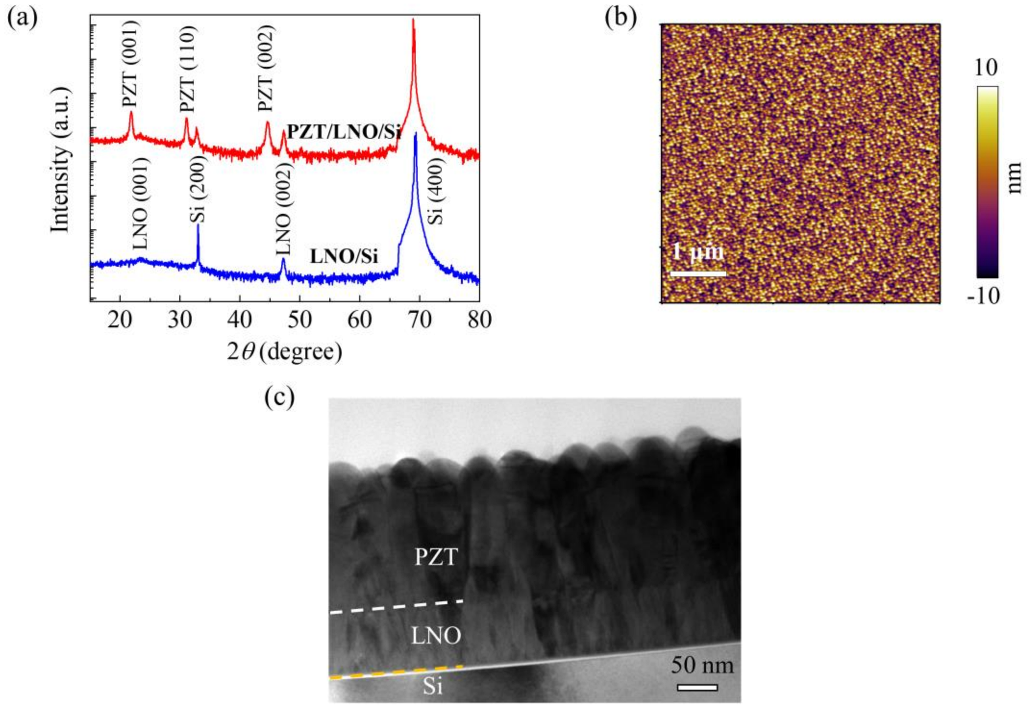

Figure S1. Structural and morphological characterizations of the PZT/LNO/Si film (related to Figure 1). (a) XRD θ-2θ diffraction patterns of a PZT/LNO/Si film and a LNO/Si film (control sample). (b) AFM topography image and (c) cross-sectional TEM image of the PZT/LNO/Si film.

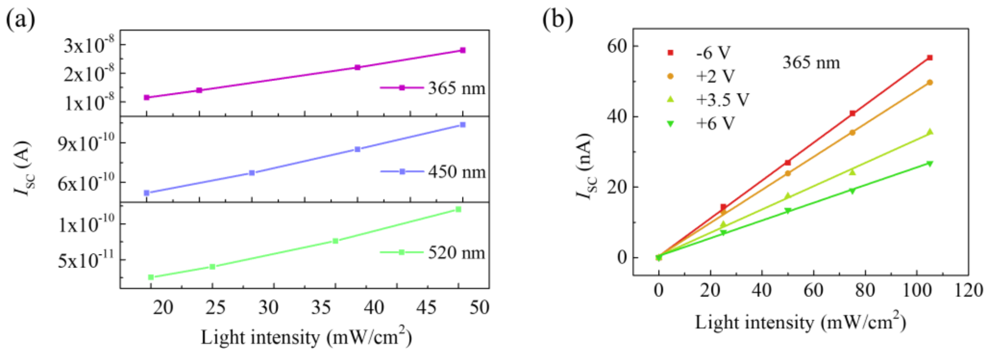

Figure S2. Effects of light wavelength and intensity on photocurrent (related to Figure 1). Photocurrent versus light intensity (a) at different light wavelengths in the P_{up} state and (b) in different polarization states under the illumination of the 365 nm UV light. In b, the polarization states were set by applying -6 V, +2 V, +3.5 V, and +6 V pulses sequentially.

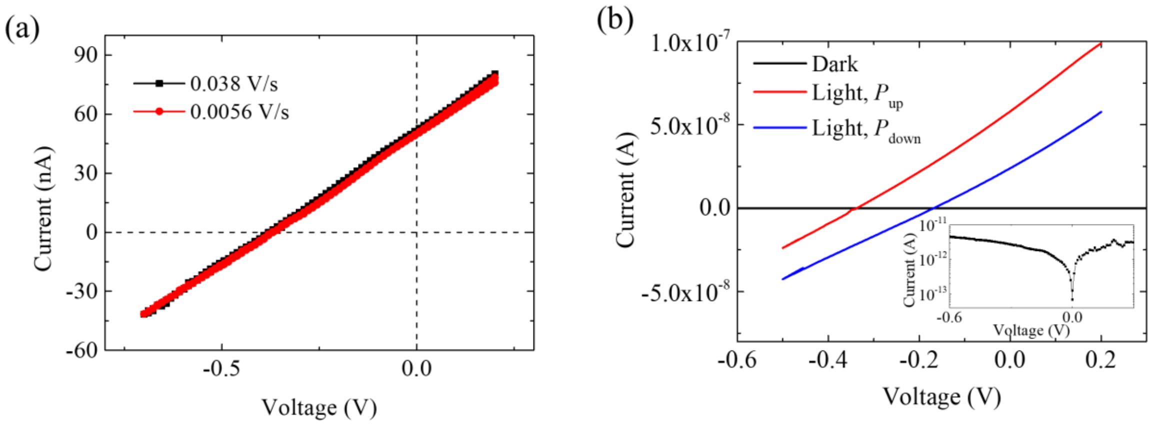

Figure S3. Demonstration of steady photocurrent and comparison between photocurrent and dark current (related to Figure 1). (a) Illuminated *I*-*V* characteristics measured in the P_{up} state with different voltage sweeping speeds. (b) Comparison between the illuminated and dark *I*-*V* characteristics. Only the dark *I*-*V* characteristics the P_{up} state are shown, because the difference between the P_{up} and P_{down} state is small (see Figure S10).

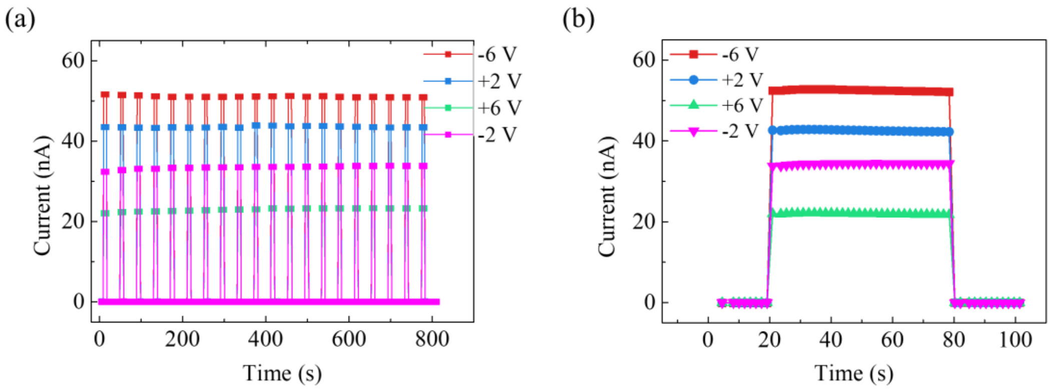

Figure S4. Stability of photovoltaic response (related to Figure 1). (a) Multi-cycle and (b) one-cycle time-dependent photocurrents (read at 0 V) of the Pt/PZT/LNO FePV device measured sequentially after applying -6 V, +2 V, +6 V and -2 V pulses to set different polarization states. In a, the light is switched ON and OFF alternately with 6 and 30 seconds for the ON and OFF periods, respectively. In b, the light is switched ON for an elongated time of ~60 seconds.

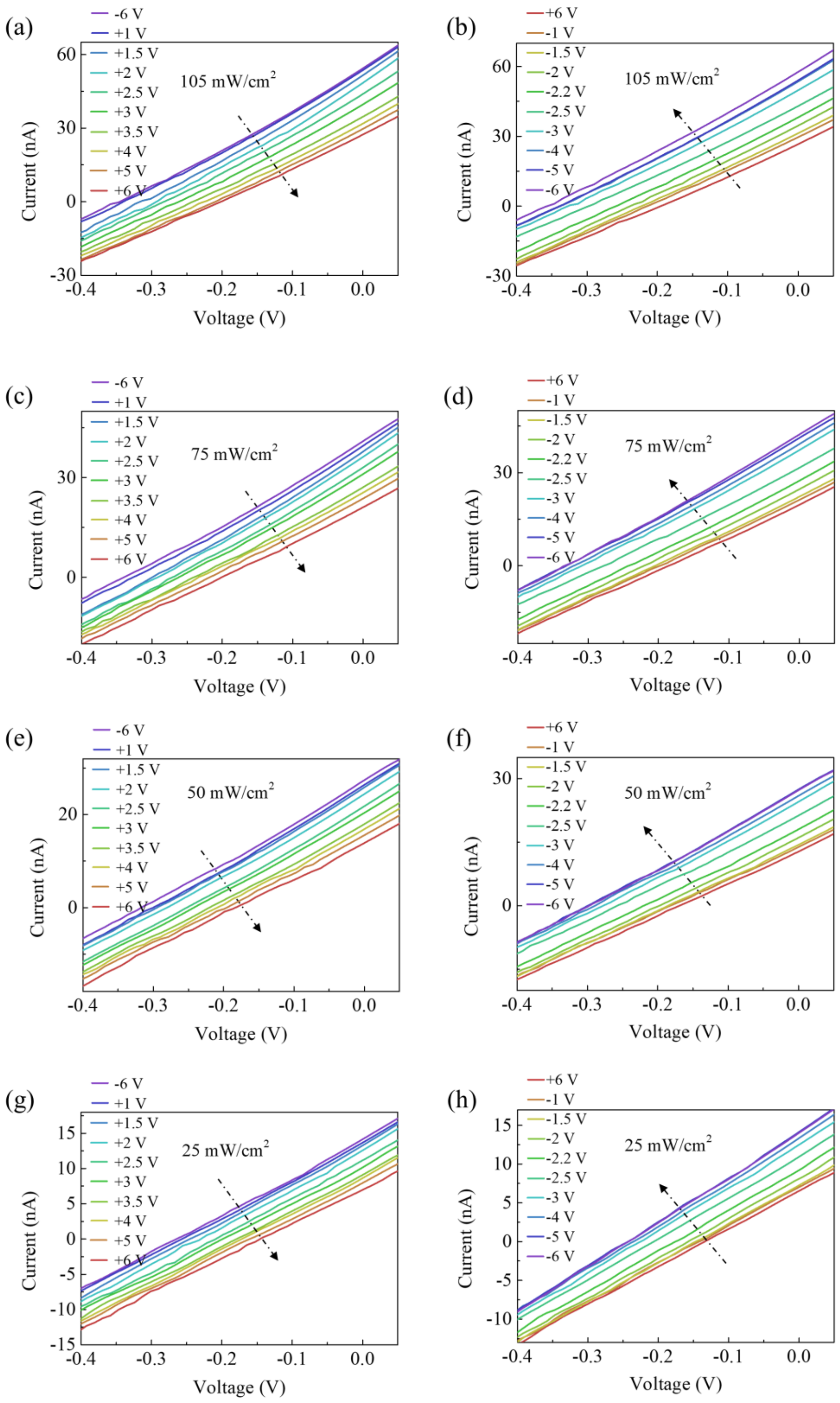

Figure S5. Effect of light intensity on illuminated *I-V* curves (related to Figure 1). Illuminated *I-V* characteristics measured after applying (a,c,e,g) positive pulses from +1 V to +6 V (starting from the initial -6 V pulse-written state) and (b,d,f,h) negative pulses from -1 V

to -6 V (starting from the initial +6 V pulse-written state) at the light intensities of (a,b) 105, (c,d) 75, (e,f) 50, and (g,h) 25 mW/cm^2. Note that the device used here is different from that used for measuring Figure 1 (main text); therefore, the two sets of illuminated *I-V* curves at 105 mW/cm^2 shown here and in Figure 1 are not exactly the same.

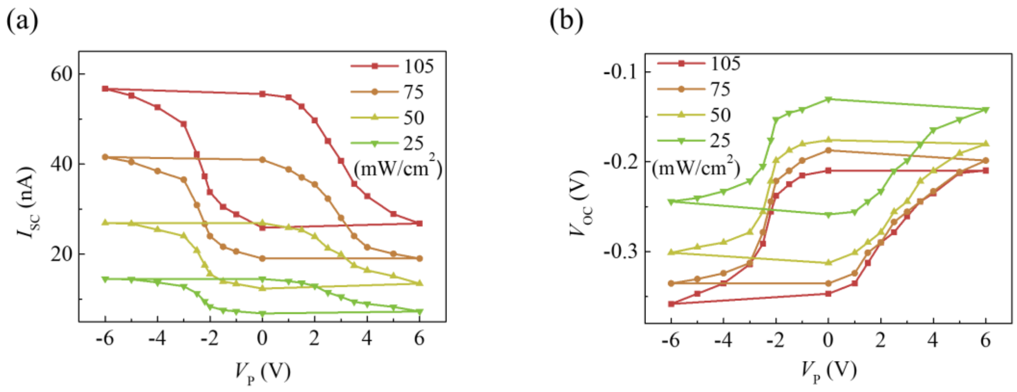

Figure S6. Effect of light intensity on the hysteretic evolutions of I_{SC} and V_{OC} (related to Figure 1). Evolutions of (a) I_{SC} and (b) V_{OC} as a function of pulse amplitude (V_p) at different light intensities. Note that the device used here is different from that used for measuring Figure 1 (main text); therefore, the two sets of the I_{SC} and V_{OC} values at 105 mW/cm^2 shown here and in Figure 1 are not exactly the same.

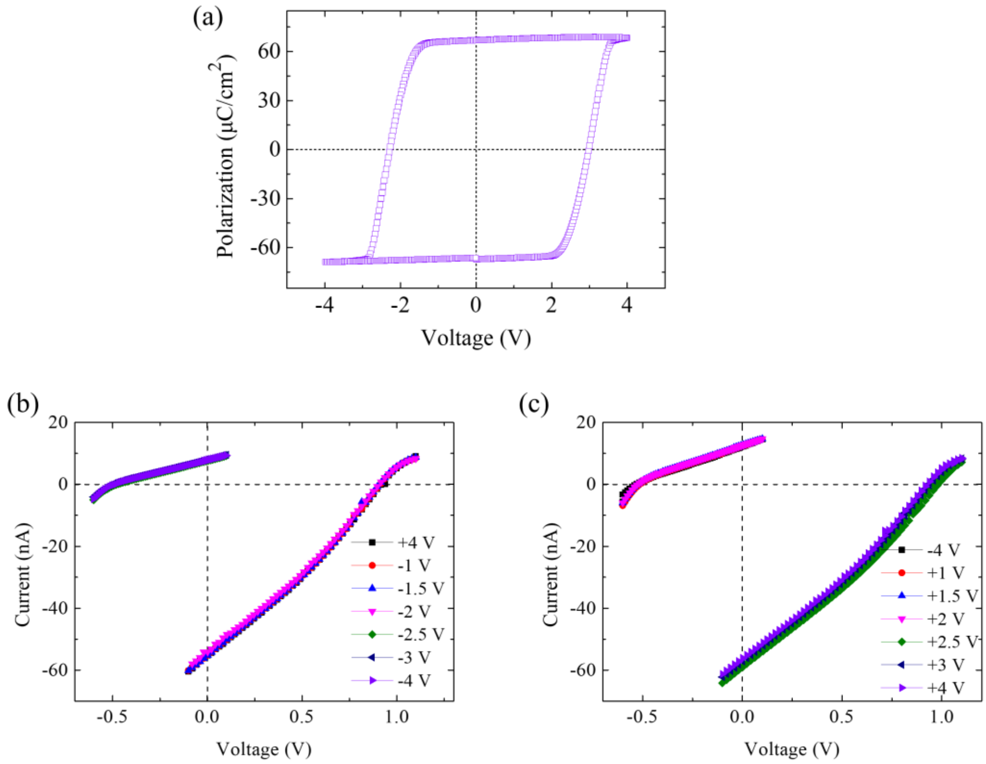

Figure S7. *P-V* hysteresis loop and two bistable photoresponsive states in the epitaxial PZT film (related to Figure 1). (a) Typical *P-V* hysteresis loop of an epitaxial PZT film sandwiched between Pt and $SrRuO_3$ electrodes grown on a $SrTiO_3$ substrate. Illuminated *I-V* characteristics measured after applying (b) negative pulses from -1 V to -4 V (starting from the initial +4 V pulse-written state) and (c) positive pulses from +1 V to +4 V (starting from the initial -4 V pulse-written state).

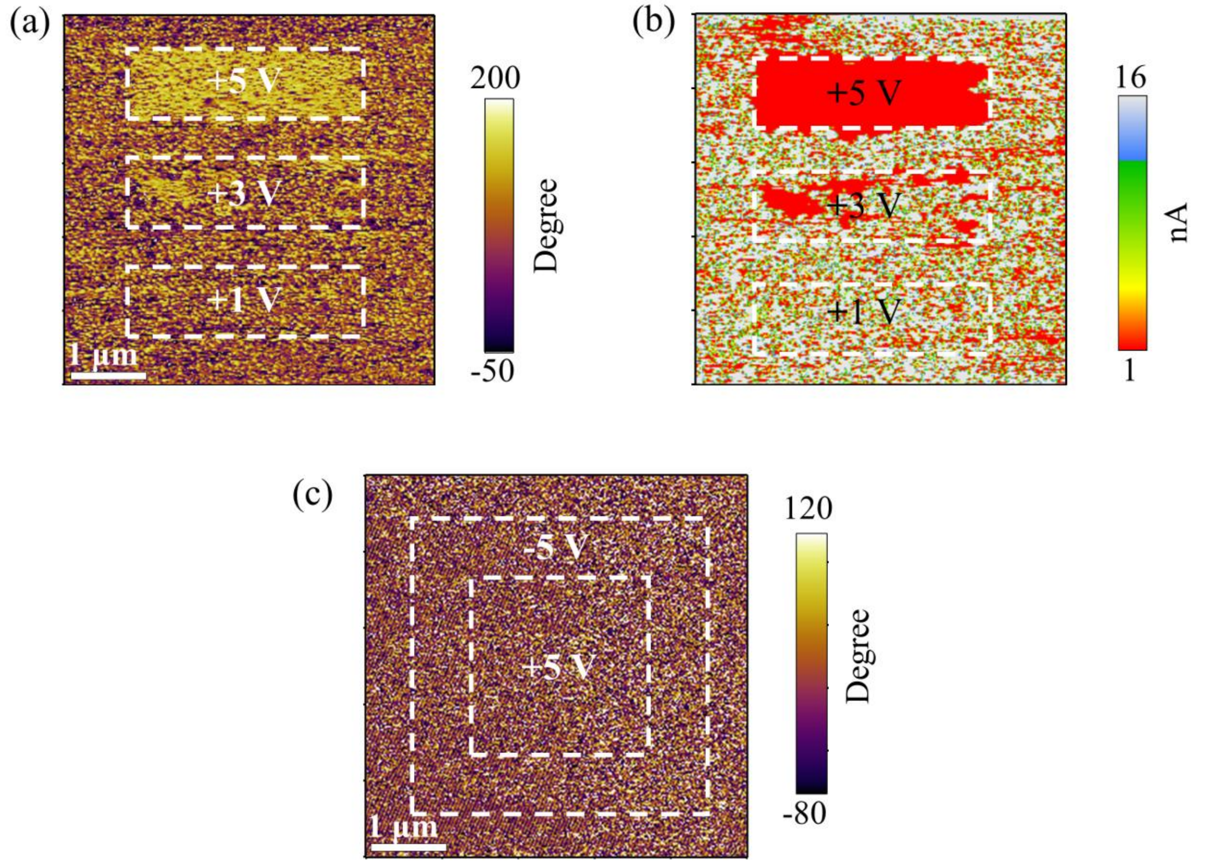

Figure S8. Microscopic observations of concurrent changes in domain configuration and photocurrent (related to Figure 2). (a) PFM phase image and (b) current map under illumination measured after writing three rectangular regions (3 × 1 μm^2) with tip biases of +5 V, +3 V, and +1 V. Note that the measurements in a and b were conducted on the Pt electrode-capped PZT film. (c) PFM phase image taken from a Pt electrode-capped SiO_2/n^{++}-Si control sample.

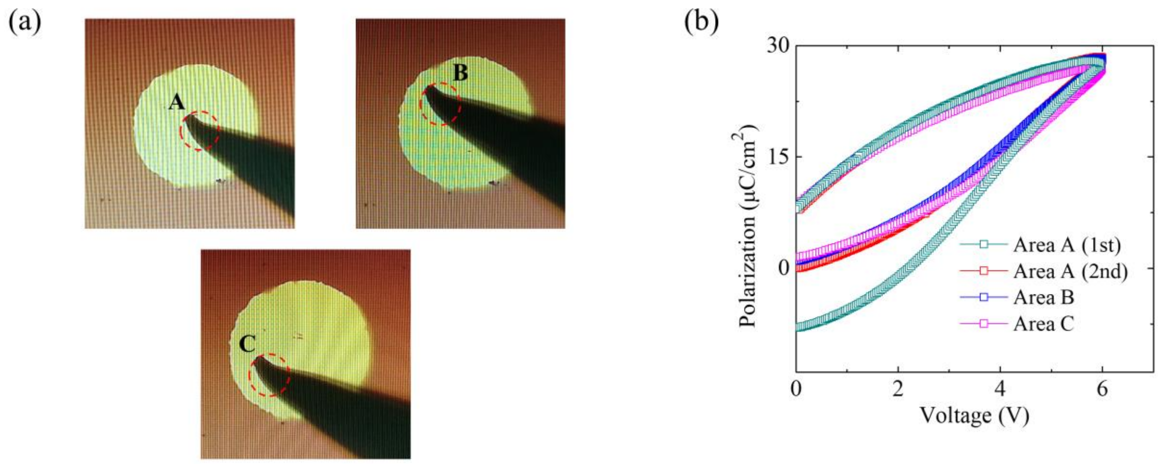

Figure S9. Polarization switching behavior with the tungsten tip-electrode contact (related to Figure 1 and Figure 2). (a) Photographs of the tungsten tip located in the different regions of the Pt electrode. (b) Monopolar *P*-*V* loops measured in the regions marked in a.

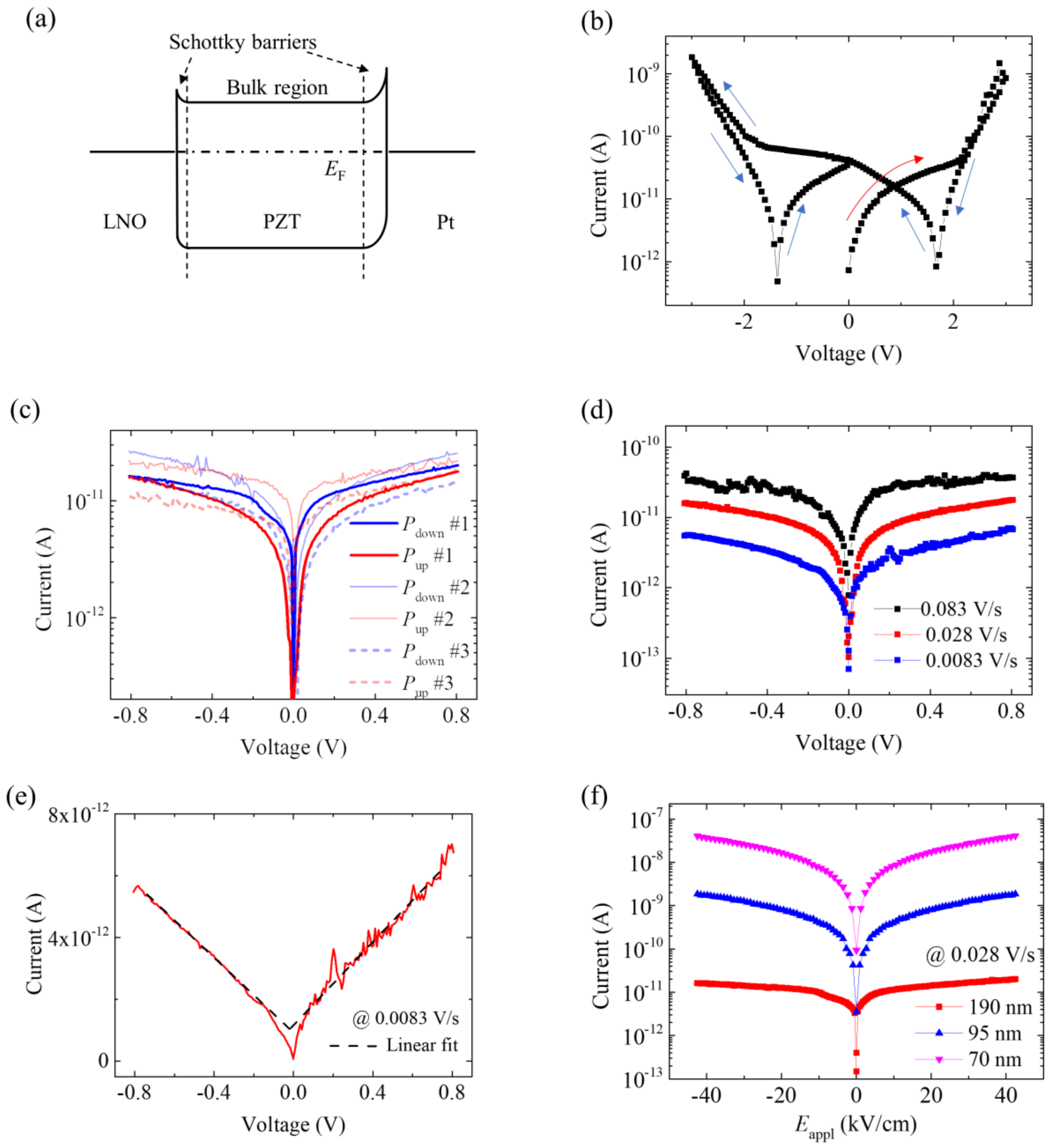

Figure S10. Conduction and photovoltaic mechanisms in the FePV device (related to Figure 1). (a) Schematic energy band diagram of the Pt/PZT/LNO device. (b) Dark *I*-*V* characteristics measured in the voltage range of +3 V to -3 V at a voltage sweeping rate of 0.06 V/s. The voltage sweeping sequence is indicated by the arrows, and the red arrow indicates the first step. (c) Dark *I*-*V* characteristics measured in the P_{up} and P_{down} states (set by -6 V and +6 V pulses, respectively) for different cycles. (d) Dark *I*-*V* characteristics measured in the P_{up}

state at different voltage sweeping rates. (e) Dark *I-V* characteristics plotted in linear scale. (f) Thickness-dependent dark *I-V* characteristics.

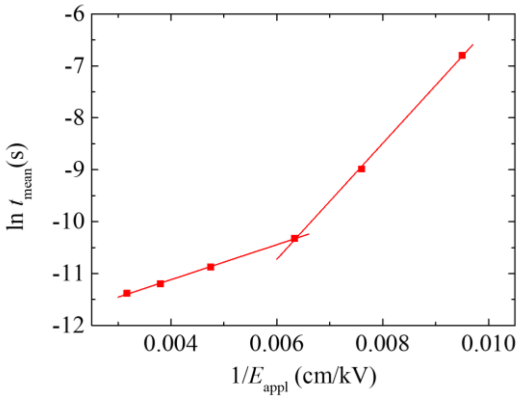

Figure S11. Analysis of domain dynamics based on the Merz's law (related to Figure 2). Relationship between mean switching time (t_{mean}) and applied electric field (E_{appl}) plotted as ln(t_{mean}) versus 1/E_{appl}.

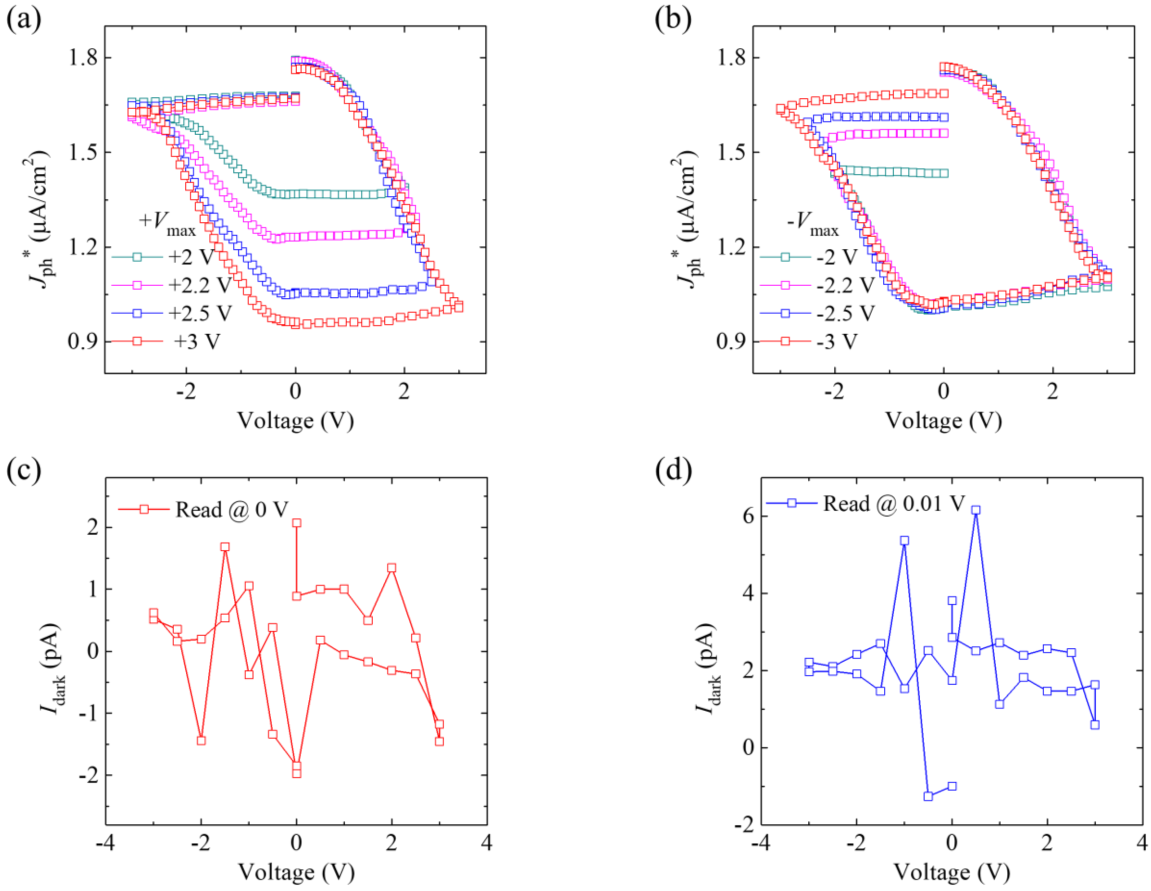

Figure S12. Comparison between photocurrent and dark current in terms of hysteretic behavior (related to Figure 3). J_{ph}^* hysteresis loops obtained with varying (a) +V_{max} and (b) -V_{max} of the triangular pulse trains at the light intensity of 25 mW/cm^2. Dark currents (I_{dark}) read at (c) 0 V and (d) 0.01 V as a function of write pulse amplitude.

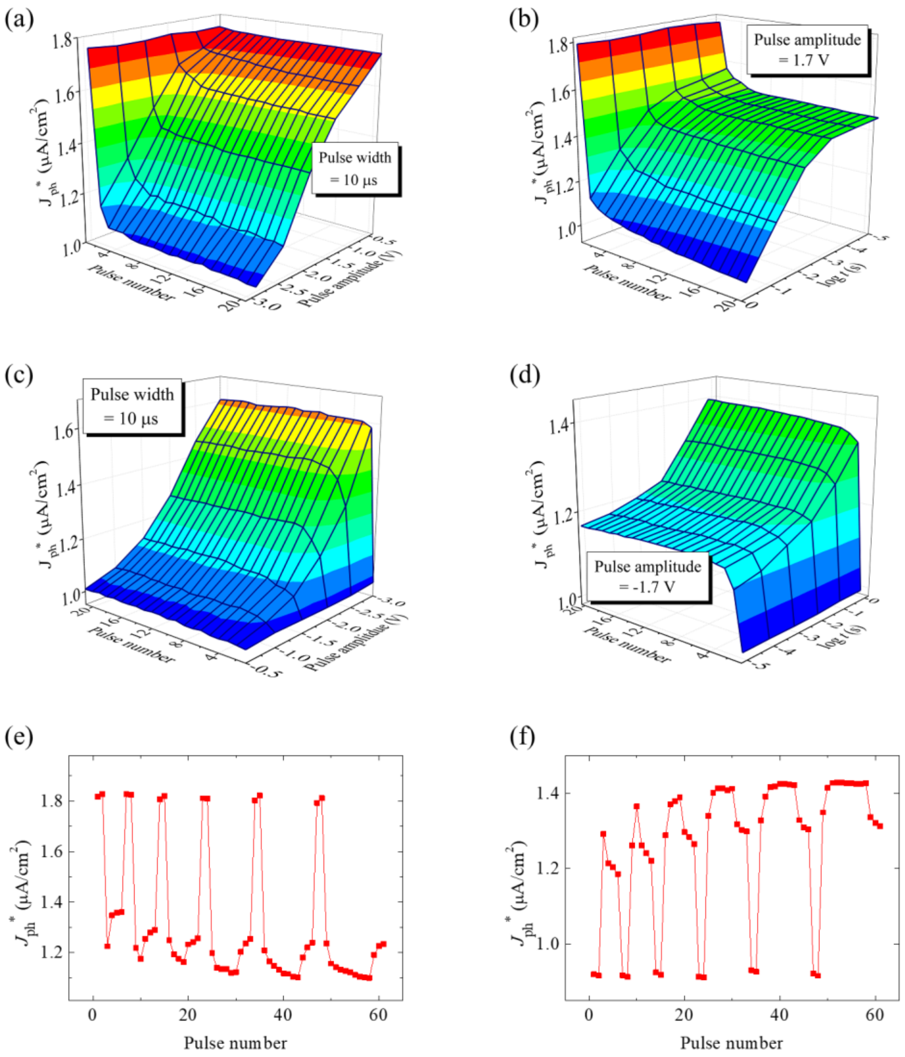

Figure S13. Continuously tunable photovoltaic response measured at the light intensity of 25 mW/cm^2 (related to Figure 3). Evolutions of J_{ph}^* measured using repeated pulses with (a,c) varying pulse number and pulse amplitude while fixing the pulse width, and (b,d) varying pulse number and pulse width while fixing the pulse amplitude. (e) Evolution of J_{ph}^* measured using the negative-positive-negative pulse train where the number of positive pulses (+2.2 V) between two negative pulse groups is varied. (f) Evolution of J_{ph}^* measured using the positive-negative-positive pulse train where the number of negative pulses (-2.2 V) between two positive pulse groups is varied. All the J_{ph}^* data were measured at the light intensity of 25 mW/cm^2.

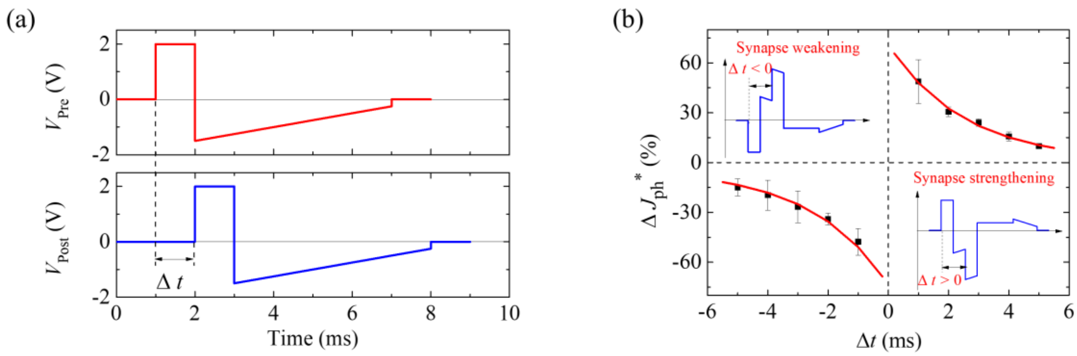

Figure S14. STDP behavior (related to Figure 4). (a) Voltage waveforms of pre- and post-synaptic spikes. (b) STDP characteristics of the Pt/PZT/LNO FePV synapse. Inset in b shows the superposed waveforms of the pre- and post-synaptic spikes.

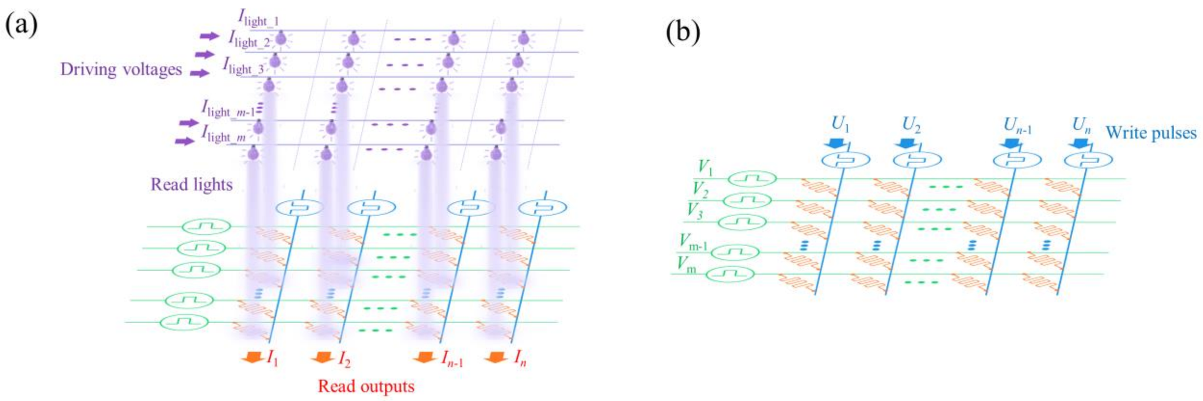

Figure S15. FePV synapse-based neural network (related to Figure 4). Schematics illustrating the (a) read and (b) write operations of the FePV synapse-based neural network.

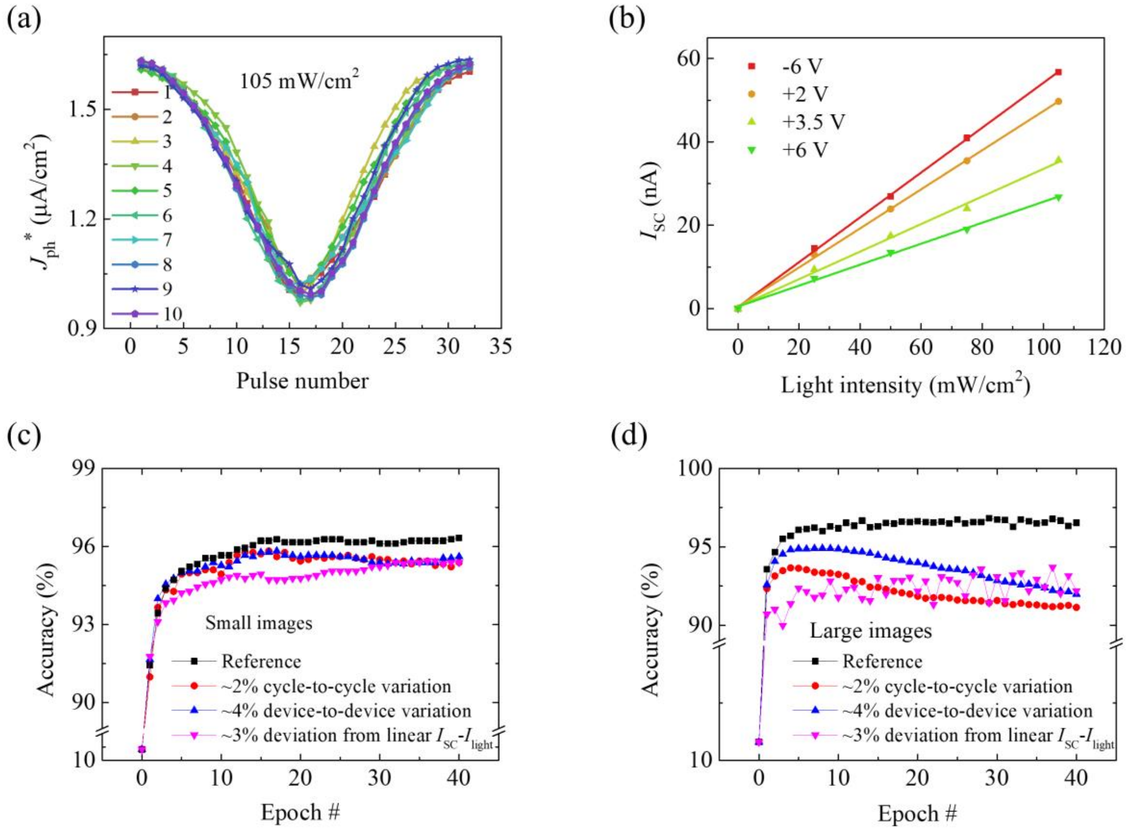

Figure S16. Effects of non-ideal factors on the simulated accuracy (related to Figure 4). (a) One-cycle LTP/LTD characteristics of 10 different FePV devices (applied pulses are the same as those in Figure 4a in the main text). (b) Photocurrent versus light intensity in different polarization states under the illumination of the 365 nm UV light. Effects of cycle-to-cycle variation, device-to-device variation, and realistic dependence of photocurrent on light intensity on the accuracies for recognizing (c) small and (d) large images. In c and d, "Reference" denotes the case where the cycle-to-cycle and device-to-device variations are zero and the dependence of photocurrent on light intensity is ideally linear.

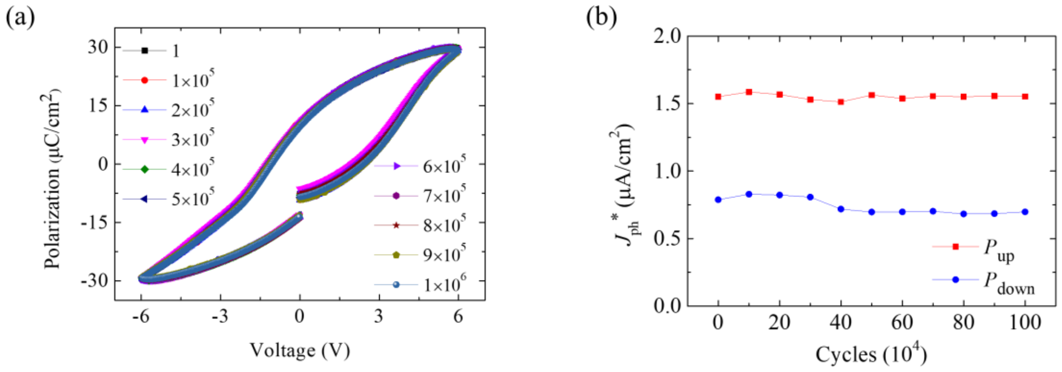

Figure S17. Endurance characteristics (related to Figure 5). Evolutions of (a) *P*-*V* hysteresis loops and (b) J_{ph}^{*} in the P_{up} and P_{down} states with increasing the number of switching cycles.

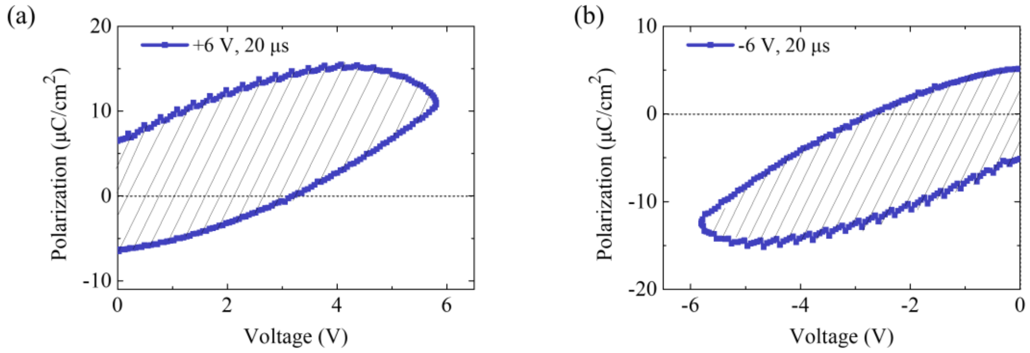

Figure S18. *P*-*V* loops for the energy consumption estimation (related to Figure 5). Typical monopolar *P*-*V* hysteresis loops measured with (a) +6 V/20 μs and (b) -6V/20 μs pulses.

Table S1. Comparison of performances of neural networks based on different synaptic devices (related to Figure 4).

Device structure	Accuracy (%)	Dataset	Operating principle
Ag/BTO/NSTO (Ma et al., 2020)	~90	MNIST	Ferroelectric domain switching
Pt/BTO/SNTO (Li et al., 2019)	96.5	ORHD[a]	Ferroelectric domain switching
	96.4	MNIST	
α -MoO_3 transistor (Yang et al., 2018)	94.1	ORHD	Li ions intercalation
	87.3	MNIST	
$Si_{0.9}/Ge_{0.1}$ (Choi et al., 2018)	95.1	MNIST	Ag ion filament
PEI/PEDOT:PSS (van de Burgt et al., 2017)	93.0	SFC[b]	Proton doping
	97.0	MNIST	
BM-SFO transistor (Ge et al., 2019)	95.2	ORHD	Phase transformation
	92.7	MNIST	
VO_2 transistor (Ge et al., 2020)	94.0	ORHD	Phase transformation
	91.0	MNIST	
Li-ion transistor (Fuller et al., 2017)	97.5	SFC	intercalation of Li-ion dopants
	93.0	MNIST	
Pt/PZT/LNO (this work)	95.4	ORHD	Ferroelectric domain switching
	93.7	MNIST	

[a)]ORHD: Optical Recognition of Handwritten Digits

[b)]SFC: Sandia file classification

Transparent Methods

1. Device Fabrication

Stoichiometric $LaNiO_3$ (LNO) and nonstoichiometric $Pb(Zr_{0.2}Ti_{0.8})O_3$ (PZT) with 15% Pb excess ceramic targets with a purity of 99.99% were bought from the Hefei Kejing Materials Technology Co., Ltd, which were used for the pulsed laser deposition (PLD) of polycrystalline LNO and PZT thin films on silicon (100) substrates, respectively. Our PLD system mainly consisted of a KrF (λ = 248 nm) excimer laser source (Coherent COMPexPro 205) and a customized chamber (TSST HE-B01 and TE-R08). The ~80 nm thick LNO bottom electrode layers were first deposited on the silicon substrates at 660 °C under an oxygen pressure of 15 Pa. Subsequently, the ~190 nm thick PZT layers were grown on top of the LNO layers at a lower temperature of 600 °C with the same oxygen pressure. For the LNO and PZT depositions, different laser energy densities of ~1.2 and ~0.9 J/cm^2 were used, respectively, while the laser pulse frequency was kept at the same 5 Hz. In addition, epitaxial PZT films, as control samples, were deposited on the $SrRuO_3$ (SRO)-buffered $SrTiO_3$ (001) substrates. The growth temperatures of the epitaxial PZT and SRO films were 615 and 680 °C, respectively, while the same oxygen pressure of 15 Pa was used for both films. The Pt top electrodes of ~200 μm in diameter and ~10 nm in thickness were *ex situ* deposited on the films by PLD through a shadow mask at room temperature and under vacuum.

Note that all the film depositions were conducted in our lab, while previous studies (Schatz et al., 2017; Yu et al., 2007; Kim et al., 2000) were referred to for optimizing the deposition parameters of the polycrystalline PZT films on silicon substrates.

2. Characterizations

The phases and crystal structures of the films were examined by X-ray diffraction (XRD) (X'Pert PRO, PANalytical). The microstructures of the films were characterized using a Tecnai

G2-F20 transmission electron microscopy (TEM) system operated at 200 kV. The film morphology, domain switching, and current mapping were studied by atomic force microscopy (AFM), piezoresponse force microscopy (PFM), and conductive AFM (C-AFM), respectively. The AFM, PFM, and C-AFM measurements were performed on a commercial atomic force microscope (Cypher, Asylum Research) with Pt-coated silicon tips (EFM Arrow, Nanoworld).

3. *Electrical Measurements*

The polarization-voltage (*P-V*) hysteresis loop, positive-up negative-down (PUND), and endurance tests were conducted with a ferroelectric workstation (Precision Multiferroic, Radiant). The current-voltage (*I-V*) characteristics were measured using a SourceMeter (6430, Keithley) with an automatic voltage sweeping program. Both the ferroelectric workstation and SourceMeter can generate electrical pulses with various amplitudes and widths (ranging from 10 μs to 1 s). For the photovoltaic measurements, an ultraviolet light-emitting diode (LED) with tunable light intensities and a wavelength of 365 nm was mainly used as the light source. Unless otherwise specified, the electrical pulse interval was fixed at 1 s, and the light intensity was fixed at 105 mW/cm^2. When measuring the illuminated *I-V* characteristics, the light was switched on ~10 seconds before the measurement to exclude the contribution from the transient photocurrent response, and a sufficiently low voltage sweeping speed (~0.038 V/s) was used to record the steady current (see Figure S3a).

4. *Neural Network Simulations*

The neural network simulations were conducted using a CrossSim simulator (van de Burgt et al., 2017; Fuller et al., 2017) based on the back-propagation algorithm. The simulator used a three-layer (one hidden layer) neural network, and each synaptic weight matrix between two neuron layers was modeled as a crossbar. The crossbar performed two matrix operations: vector-matrix multiply and parallel rank one outer product update. The numerical weights in a crossbar were mapped directly onto the experimental J_{ph}^* values measured in the

potentiation/depression cycling test (see Figure 4a in the main text), and thus the numbers of weight levels were 16 for both the potentiation and depression processes. The number of neurons and synapses used in the image recognition simulations were dependent on the image datasets. For small images (8 × 8 pixels), 64, 36, and 10 neurons were used in the input, hidden, and output layers, respectively. The numbers of synapses in the two crossbars were therefore 2304 (64 × 36) and 360 (36 × 10), respectively. For large images (28 × 28 pixels), a network size of 784 × 300 × 10 was used. When training a neural network, 3823 and 60000 examples were used for small and large images, respectively. Afterwards, the accuracy of the neural network was tested with 1797 (10000) examples for small (large) images. The learning rates for the simulations of small and large image recognitions were optimized to be 0.15 and 0.025, respectively.

5. *Data Analysis for Figure S1*

Figure S1a shows the XRD θ-2θ diffraction patterns of a PZT/LNO/Si bilayer film and a LNO/Si film, with the latter as the control sample. The (001) and (110) peaks from PZT are clearly identified, indicating that polycrystalline PZT with a perovskite phase is formed. There are no peaks from impurity phases, demonstrating the phase purity of the PZT film. Figure S1b displays that the polycrystalline PZT film exhibits a granular surface, with a relatively small roughness of ~4 nm. The cross-sectional TEM image, as shown in Figure S1c, demonstrates that the PZT film is tightly adhered to the LNO layer with a sharp interface. Additionally, the PZT film exhibits columnar grains, which are ~190 nm in height and ~50 nm in diameter.

6. *Data Analysis for Figure S2*

Figure S2a shows that at the same light intensity the 365 nm UV light produces the highest photocurrent, which is consistent with the fact that PZT has a relatively wide bandgap of ~3.6 eV (Tan et al., 2019). In addition, as shown in Figure S2b, in all the different polarization states the photocurrent scales almost linearly with the light intensity. As seen from the above results,

the 365 nm UV light with the intensity of 105 mW/cm^2 is a suitable illumination condition. Therefore, this illumination condition was mainly used in the main text.

7. *Data Analysis for Figure S3*

As shown in Figure S3a, although the voltage sweeping speed decreases from ~0.038 V/s to ~0.0056 V/s, the photovoltaic *I-V* curve remains almost unchanged, demonstrating that the current values measured at ~0.038 V/s are already steady ones. In addition, the light was switched ON ~10 seconds before the *I-V* measurement to exclude the contribution from the transient photocurrent response. Figure S3b shows that the photocurrent is orders of magnitude larger than the dark current at low voltage. In addition, measuring the photocurrent at zero voltage has technically eliminated the contribution from the dark current.

8. *Data Analysis for Figure S4*

Applications of -6 V and +6 V write pulses (pulse width: 1 ms) result in the fully upward and downward polarization states, respectively. On the other hand, applications of +2 V pulse (after the -6 V pulse) and -2 V pulse (after the 6 V pulse) (pulse width: 10 μs) result in two intermediate polarization states. Figure S4 show that the photocurrents measured in all these states are quite stable and reproducible, indicating the nonvolatility of the photoresponsive states. In Figure S4b, no spike is observed right after the light is switched ON, probably because the photocurrent response time in our polycrystalline PZT film is sufficiently short (less than ~1.8 seconds, i.e., the time resolution used in the photocurrent-time measurement). This allows us to measure the steady current when performing the photovoltaic *I-V* measurement.

9. *Data Analysis for Figure S5 and Figure S6*

Figure S5 shows that the successive shift of the illuminated *I-V* curve with applied write pulse occurs at the different light intensities. In addition, both I_{SC} and V_{OC} show hysteretic evolutions with varying pulse amplitude at different light intensities (see Figure S6). The I_{SC}

and V_{OC} values in the same polarization state increase as the light intensity increases, indicating that the I_{SC} and V_{OC} are light-induced. The above results well demonstrate the phenomenon of polarization-modulated switchable photovoltaic response.

10. Data Analysis for Figure S7

Figure S7a shows the square *P-V* hysteresis loop observed in the epitaxial PZT film, indicating that two typical bistable polarization states exist and the switching between these two states is abrupt. Consequently, there are two bistable photoresponsive states and the switching between them is also observed to be abrupt, as shown in Figure S7b,c.

11. Data Analysis for Figure S8

Three rectangular regions (3×1 μm^2) were written with tip biases of +5 V, +3 V and +1 V, respectively, and then PFM and C-AFM images were measured. The C-AFM measurement was conducted with the sample bias but zero sample bias was indeed applied for measuring the photocurrent. The sign of the measured photocurrent was reversed because the current flowing from top to bottom was defined to be positive.

Figure S8a displays that almost all domains in the +5 V-written region and partial domains in the +3 V-written region are switched downward, while the domains in the +1 V-written region remain almost unchanged. The different degrees of domain switching lead to different changes in photocurrent. As shown in Figure S8b, the photocurrents in the whole +5 V-written region are decreased significantly, while in the +3 V-written region only the photocurrents in the parts where domains are switched downward are decreased. However, the photocurrents in the +1 V-written region are similar to those in the unwritten region. The combined PFM and C-AFM results therefore confirm that the photovoltaic response is modulated by the polarization.

Note that the Pt top electrode was used to enhance the magnitude of photocurrent; otherwise, the photocurrent can hardly be detected using the AFM tip-film contact. The Pt electrode may decrease the interfacial barrier height or increase the contact area, thereby enhancing the

magnitude of photocurrent. However, the local switching of domains is observed with the Pt electrode. To understand the origin, a similar electrical writing was performed on a Pt electrode-capped SiO_2/n^{++}-Si control sample. The resultant PFM phase image shows no changes in the written areas (Figure S8c), thus excluding the surface effect-induced artifacts. We therefore think that the Pt electrode may not be an ideal conductor, because it was thin (~10 nm) and composed of nanosized grains. Therefore, electrically writing on the Pt electrode-capped PZT film using the AFM tip induces the domain switching and photocurrent change only in a local area (see Figure S8a,b). This local switching behavior with a non-ideal top electrode was reported previously (Li et al., 2017).

Indeed, for a non-ideal electrode, the tip-generated field drops as a nonlinear function of distance from the tip-electrode contact along the in-plane direction (Li et al., 2017). This means that when a voltage V is applied via the tip, the potential V_1 in the tip-electrode contact area (A_0) is larger than the potential V_2 in the area outside A_0 (note: we use this qualitative description because the exact function of the potential distribution is unknown). This non-uniform potential distribution may cause non-uniform domain switching within the electrode area. Nevertheless, when V is sufficiently large, both V_1 and V_2 exceed coercive voltage (V_c) and thus the domains in and outside the area A_0 can be switched. The total area where the domains are switched (A_s) therefore depends on the voltage V and the tip-electrode contact area A_0. For a given V, if A_0 is larger, a larger area is subjected to V_1 and thus more domains can be switched.

The AFM tip has a very small A_0 (tip radius: ~15 nm). Even if $A_s/A_0 = 100$ is assumed, the resultant radius of A_s is ~150 nm, which is still very small. Therefore, using the AFM tip to write on the electrode can cause a local switching. By contrast, the tungsten tip has a much larger A_0 of ~1.5×10^{-5} cm^2 (confirmed by checking the scratch left by tungsten tip), about ~5% of the whole electrode area. If V is sufficiently large, it could be possible that A_s becomes the whole electrode area (see the demonstration in Figure S9).

12. Data Analysis for Figure S9

For the tungsten tip-electrode contact, to verify whether A_s can be the whole electrode area under a large voltage, we first located the tungsten tip in Region A, B, and C, and applied -6 V pulses to set the polarization states of all the three regions to P_{up}. Then, the tungsten tip was located in Region A and two +6 V pulse were applied sequentially. The measured monopolar *P-V* loops are shown in Figure S9b. The first +6 V pulse switches the polarization to the downward direction and therefore an "S"-shaped loop is present. However, the loop measured by the second +6 V pulse does not exhibits an apparent "S" shape. This is because the polarization is already along the downward direction, and hence this loop is contributed mainly by non-ferroelectric factors, such as leakage current and linear dielectric response. Then, we moved the tungsten tip to Region B and C sequentially, and measured the monopolar *P-V* loop in each region by applying a +6 V pulse. The loops measured in both Region B and C are similar to that measured in Region A with the second +6 V pulse, indicating that the domains in Region B and C have already been switched to the downward direction after applying +6 V pulses in Region A. This in turn supports that applying a sufficiently large voltage via the tungsten tip-electrode contact can switch the domains in the whole electrode area.

13. Data Analysis for Figure S10

Generally, there are four FePV mechanisms accounting for the polarization modulation of photovoltaic response: bulk photovoltaic effect, domain wall model, Schottky barrier model, and depolarization field (E_{dp}) model.

The bulk photovoltaic effect often occurs in a single crystal, where the photo-generated charge carriers with asymmetric momentum distribution can exhibit collective motion along a certain direction (Fridkin et al., 2001). However, our PZT film is polycrystalline, the momentum distributions of photo-generated charge carriers may cancel each other out. Therefore, the bulk photovoltaic effect may be neglected in our polycrystalline PZT-based FePV device. In addition,

the domain walls in the polycrystalline PZT film are not aligned in parallel, which rules out the domain wall model as the major photovoltaic mechanism in our FePV device.

To check the applicability of the Schottky barrier model, the energy band alignment of the Pt/PZT/LNO heterostructure is first analyzed. Because Pt and LNO have work functions of ~5.6 eV (Yan et al., 2010) and ~4.5 eV (Yang et al., 2012), respectively, and PZT has an electron affinity of ~3.5 eV (Cagin et al., 2007), two Schottky barriers with much different heights may exist in the Pt/PZT/LNO heterostructure (see Figure S10a). If the conduction is dominated by the Schottky barriers, *I-V* curves with large asymmetry are expected. However, the asymmetry of the observed dark *I-V* curves is rather small (Figure S10b). In addition, Figure S10b and c also reveal that the switchable diode-type resistive switching behavior is absent. In Figure S10b, a small hysteresis is observed when the voltage sweeping direction is reversed and the dark current (I_{dark}) becomes zero at a non-zero voltage, both of which can be correlated with the change of the capacitive current (I_{cap}) due to the reverse of the sign of dV/dt and the change of capacitance [note: $I_{cap} = C$*(dV/dt)]. In Figure S10c, I_{dark} randomly varies from cycle to cycle and it does not show a variation trend with respect to the polarization state. This can be well explained by the randomness of I_{cap}, which may originate from the random capacitance variation caused by the pulse writing-induced charge injection and redistribution. The contribution from I_{cap} to I_{dark} can be evidenced by measuring the dark *I-V* characteristics at different voltage sweeping rates (i.e., dV/dt). Figure S10d shows that the measured I_{dark} increases with voltage sweeping rate, confirming the contribution from I_{cap} to I_{dark}.

Therefore, the above results rule out the Schottky barrier-dominated conduction mechanism and the ferroelectric memristive effect. They also demonstrate that I_{cap} can significantly contribute to the measured $I_{dark,}$ particularly when the current level is low.

While the polycrystalline PZT film has a thickness of ~190 nm, a large bulk region exists (Figure S10a) and may greatly influence the conduction and associated FePV effect. Indeed, the dark *I-V* curves can be well fitted to the Ohmic model (see Figure S10e), suggesting that

the conduction mechanism (in the voltage region of -0.8 V to 0.8 V) is bulk-limited (note: to minimize the involvement of I_{cap}, the dark *I-V* curve measured at a very slow rate of 0.0083 V/s was used for fitting). Moreover, I_{dark} decreases with increasing film thickness (see Figure S10f), agreeing well with bulk-limited conduction behavior. For our polycrystalline PZT film, therefore, the conduction mechanism is bulk-limited and consequently a bulk mechanism should also be responsible for the FePV effect.

The only left bulk mechanism for the FePV effect is the E_{dp} model. In this model, a switchable E_{dp} and an unswitchable internal bias field (E_{int}) may be considered as the driving forces for the photovoltaic effect. E_{dp} arises from the incomplete screening of polarization, while E_{int} may be formed due to stress gradients (Zhou et al., 2005), asymmetric electrodes (Lee et al., 1998), and non-uniformly distributed space charges (Tagantsev et al., 2004). How E_{dp} and E_{int} modulate the photovoltaic response is explained in the main text.

14. Data Analysis for Figure S11

The relationship between t_{mean} and pulse amplitude can be described using the Merz's law:

$$t_{mean} \propto \exp\left(\alpha / E_{appl}\right), \quad \text{(S1)}$$

where α is the activation field and E_{appl} is the applied electric field (i.e., the pulse amplitude divided by the film thickness). As shown in Figure S11, the ln(t_{mean})-1/E_{appl} curve exhibits two linear regions with different slopes. The α values in the low-E_{appl} and high-E_{appl} regions are ~1100 and ~340 kV/cm, respectively. These α values are consistent with those reported for the PZT films (So et al., 2005; Jo et al., 2007). In addition, the change of α as E_{appl} increases was also observed previously (So et al., 2005), which may be attributed to the different nucleation rates in the low-E_{appl} and high-E_{appl} regions.

15. Data Analysis for Figure S12

Figure S12a,b show the evolutions of J_{ph}^* with the pulse amplitudes measured at the light intensity of 25 mW/cm^2 using the triangular pulse trains. The J_{ph}^* evolutions form the hysteresis loops and multiple intermediate J_{ph}^* levels can be obtained, similar to those measured at the light intensity of 105 mW/cm^2 (Figure 3b,c in the main text).

When no illumination is applied, the dark currents (I_{dark}) read at 0 V are simply noisy signals and no variation trend of I_{dark} is observed as the pulse amplitude varies (Figure S12c). We also conducted the same measurement with the I_{dark} read at 0.01 V. Again, I_{dark} does not show hysteretic evolution with varying pulse amplitude (Figure S12d). This is consistent with the absence of ferroelectric memristive effect in our polycrystalline PZT film (see Figure S10 and related discussion).

In addition, the variations of J_{ph}^* as a function of pulse amplitude, width, and history were measured at the light intensity of 25 mW/cm^2. The results are shown in Figure S13, which are basically similar to those measured at the light intensity of 105 mW/cm^2 (Figure 3d-i in the main text).

16. Data Analysis for Figure S15

In the read operation, no read voltages are applied along the rows (see Figure S15a). Because for our FePV synapses J_{ph}^* exhibits a highly linear dependence on the light intensity (see Figure S2), the pixel values of the input images are thus encoded as the light intensities. Consequently, the output photocurrent along the jth column is

$$I_j = \sum_{i=1}^{m} J^*_{\mathrm{ph}\_ij} \times \left[I_{\mathrm{light}\_i} / (1\ \mathrm{mW/cm^2}) \right] \times A \qquad \text{(S2)}$$

where I_{light_i}/(1 mW/cm^2) is the normalized light intensity applied along the ith row and A is the device area of a single synapse. Eq. (S2) indicates that a parallel read operation or a vector-matrix multiply is realized with the FePV devices used as the synapses. To represent the negative weights, a bias row and column can be added, which are not drawn here.

In the write operation, the programming pulses (U_j) are applied along the columns corresponding to the selected synapses while the corresponding rows are grounded (V_i = 0) (here, i and j are the row and column indices of the selected synapses). The number and amplitude of the programming pulses are calculated based on the desired weight updates obtained from the back-propagation algorithm. In this way, the weights of all the selected synapses can be updated appropriately. For the unselected rows and columns, the half-voltage scheme may be employed to reduce the write disturbance.

17. Data Analysis for Figure S16

First, the accuracies of the neural networks with and without considering the cycle-to-cycle variation were computed. As the cycle-to-cycle variation changes from 0 to the experimental value of ~2%, the accuracy decreases from 96.3% (@ 40th epoch) to 95.8% (@ 16th epoch) for recognizing small images (Figure S16c), while that decreases from 96.8% (@ 29th epoch) to 93.7% (@ 4th epoch) for recognizing large images (Figure S16d). Then, the LTP/LTD characteristics of 10 different devices were measured and are shown in Figure S16a, based on which the device-to-device variation is estimated as ~4%. Figure S16c,d compares the accuracies of the neural networks with and without considering the device-to-device variation. As the device-to-device variation changes from 0 to ~4%, the accuracy decreases from 96.3% (@ 40th epoch) to 95.8% (@ 17th epoch) for recognizing small images (Figure S16c), while that decreases from 96.8% (@ 29th epoch) to 94.9% (@ 8th epoch) for recognizing large images (Figure S16d). Similar degradations of neural network performance with the cycle-to-cycle and device-to-device variations were reported in Ref. (Ma et al., 2020).

We also considered the factor of the realistic dependence of photocurrent (I_{SC}) on light intensity (I_{light}). As shown in Figure S16b, the realistic dependence deviates from the ideal linear dependence, and the deviation is ~3%. This means that $J_{ph}^* = I_{SC}/A/[I_{light}/(1\ mW/cm^2)]$, where A is the device area and $I_{light}/(1\ mW/cm^2)$ is the normalized light intensity, is not a constant at

different I_{light}. In terms of the neural network performance, the accuracy obtained with realistic dependence is ~0.9% (~3.1%) lower than that obtained with the ideal linear dependence for recognizing small (large) images (See Figure S16c,d).

18. Data Analysis for Figure S18

The write energy can be calculated from the area enclosed by the loop and the vertical axis (Xu et al., 2017). Based on Figure S18a,b, the write energies are calculated to be ~18 nJ for potentiation and ~23 nJ for depression.

References

Cagin, E., Chen, D.Y., Siddiqui, J.J., and Phillips, J.D. (2007). Hysteretic metal-ferroelectric-semiconductor capacitors based on PZT/ZnO heterostructures. J. Phys. D: Appl. Phys. *40,* 2430.

Choi, S., Tan, S.H., Li, Z., Kim, Y., Choi, C., Chen, P., Yeon, H., Yu, S., and Kim, J. (2018). SiGe Epitaxial Memory for Neuromorphic Computing with Reproducible High Performance Based on Engineered Dislocations. Nat. Mater. *17*, 335–340.

Fridkin, V. M. Bulk photovoltaic effect in noncentrosymmetric crystals. (2001). Crystallogr. Rep. *46*, 654.

Fuller, E.J., Gabaly, F.E., Léonard, F., Agarwal, S., Plimpton, S.J., J.-Gedrim, R.B., James, C.D., Marinella, M.J., and Talin, A.A. (2017). Li-Ion Synaptic Transistor for Low Power Analog Computing. Adv. Mater. *29*, 1604310.

Ge, C., Li, G., Zhou, Q.L., Du, J.Y., Guo, E.J., He, M., Wang, C., Yang, G.Z., and Jin, K.J. (2020). Gating-Induced Reversible H_xVO_2 Phase Transformations for Neuromorphic Computing. Nano Energy *67*, 104268.

Ge, C., Liu, C., Zhou, Q., Zhang, Q., Du, J., Li, J., Wang, C., Gu, L., Yang, G., and Jin, K. (2019). A Ferrite Synaptic Transistor with Topotactic Transformation. Adv. Mater. *31,* 1900379.

Jo, J.Y., Han, H.S., Yoon, J.-G., Song, T.K., Kim, S.-H., and Noh, T.W. (2007). Domain Switching Kinetics in Disordered Ferroelectric Thin Films. Phys. Rev. Lett. *99*, 267602.

Kim, S.S., Kang, T.S., and Je, J.H. (2000). Structures and properties of (001)-oriented $Pb(Zr,Ti)O_3$ films on $LaNiO_3$/Si(001) substrates by pulsed laser deposition. J. Mater. Res. *15,* 2881.

Lee, J., Choi, C.H., Park, B.H., Noh, T.W., and Lee, J.K. (1998). Built-in voltages and asymmetric polarization switching in $Pb(Zr,Ti)O_3$ thin film capacitors. Appl. Phys. Lett. *72,* 3380.

Li, J., Ge, C., Du, J., Wang, C., Yang, G., and Jin, K. (2019). Reproducible Ultrathin Ferroelectric Domain Switching for High-Performance Neuromorphic Computing. Adv. Mater. *32*, 1905764.

Li, T., Sharma, P., Lipatov, A., Lee, H., Lee, J.-W., Zhuravlev, M.Y., Paudel, T.R., Genenko, Y.A., Eom, C.-B., Tsymbal, E.Y., et al. (2017). Polarization-Mediated Modulation of Electronic and Transport Properties of Hybrid MoS_2-$BaTiO_3$-$SrRuO_3$ Tunnel Junctions. Nano Lett. *17*, 922–927.

Ma, C., Luo, Z., Huang, W., Zhao, L., Chen, Q., Lin, Y., Liu, X., Chen, Z., Liu, C., Sun, H., et al. (2020). Sub-Nanosecond Memristor Based on Ferroelectric Tunnel Junction. Nat. Commun. *11*, 1439.

Schatz, A., Pantel, D., and Hanemann, T. (2017). Towards low-temperature deposition of piezoelectric $Pb(Zr,Ti)O_3$: Influence of pressure and temperature on the properties of pulsed laser deposited $Pb(Zr,Ti)O_3$. Thin Solid Films *636*, 680-687.

So, Y.W., Kim, D.J., Noh, T.W., Yoon, J.-G., and Song, T.K. (2005). Polarization Switching Kinetics of Epitaxial $Pb(Zr_{0.4}Ti_{0.6})O_3$ Thin Films. Appl. Phys. Lett. *86*, 092905.

Tagantsev, A.K., Stolichnov, I., Setter, N., and Cross, J.S. (2004). Nature of nonlinear imprint in ferroelectric films and long-term prediction of polarization loss in ferroelectric memories. J. Appl. Phys. *96*, 6616.

Tan, Z., Hong, L., Fan, Z., Tian, J., Zhang, L., Jiang, Y., Hou, Z., Chen, D., Qin, M., Zeng, M., et al. (2019). Thinning Ferroelectric Films for High-Efficiency Photovoltaics Based on the Schottky Barrier Effect. NPG Asia Mater. *11*, 20.

van de Burgt, Y., Lubberman, E., Fuller, E.J., Keene, S.T., Faria, G.C., Agarwal, S., Marinella, M.J., Talin, A.A., and Salleo, A. (2017). A non-volatile organic electrochemical device as a low-voltage artificial synapse for neuromorphic computing. Nat. Mater. *16*, 414-418.

Xu, B., Íñiguez, J., and Bellaiche, L. (2017). Designing lead-free antiferroelectrics for energy storage. Nat. Commun. *8*, 15682.

Yan, F., Sterianou, I., Miao, S., Reaney, I.M., Lai, M.O., and Lu, L. (2010). Multiferroic properties of $Bi(Fe_{0.5}Sc_{0.5})O_3$-$PbTiO_3$ thin films. Phys. Scr. *T139*, 014003.

Yang, C., Shang, D., Liu, N., Fuller, E.J., Agrawal, S., Talin, A.A., Li, Y., Shen, B., and Sun, Y. (2018). All-Solid-State Synaptic Transistor with Ultralow Conductance for Neuromorphic Computing. Adv. Funct. Mater. *28*, 1804170.

Yang, T.-H., Harn, Y.-W., Chiu, K.-C., Fana, C.-L., and Wu, J.-M. (2012). Promising electron field emitters composed of conducting perovskite $LaNiO_3$ shells on ZnO nanorod arrays. J. Mater. Chem. 22, 17071-17078.

Yu, Y.H., Lai, M.O., and Lu, L. (2007). Highly (100) oriented $Pb(Zr_{0.52}Ti_{0.48})O_3$/$LaNiO_3$ films grown on amorphous substrates by pulsed laser deposition. Appl. Phys. A *88*, 365–370.

Zhou, Y., Chan, H.K., Lam, C.H., and Shin, F.G. (2005). Mechanisms of imprint effect on ferroelectric thin films. J. Appl. Phys. *98*, 024111.
